# Dietary Phospholipids and Their Impact on Crustacean Physiology: Growth, Metabolism, Immunity, and Beyond

**DOI:** 10.1155/anu/8180797

**Published:** 2025-06-13

**Authors:** Hrishika Barua, Mahima Ranjan Acharjee, Stephen G. Giteru, Methila Chowdhury, Haizhou Wu, Lokesh Kumar, Mirja Kaizer Ahmmed

**Affiliations:** ^1^Department of Fishing and Post-Harvest Technology, Faculty of Fisheries, Chattogram Veterinary and Animal Sciences University, Chattogram, Bangladesh; ^2^Department of Aquaculture, Faculty of Fisheries, Chattogram Veterinary and Animal Sciences University, Chattogram, Bangladesh; ^3^Sales and Marketing Department, Alliance Group Limited, Invercargill, New Zealand; ^4^College of Animal Science and Technology, College of Veterinary Medicine, Huazhong Agricultural University, Wuhan, Hubei, China; ^5^Department of Wine, Food and Molecular Biosciences, Lincoln University, Lincoln, Christchurch, New Zealand

**Keywords:** aquaculture, crustacean nutrition, lecithin, phospholipid-enriched diet, reproduction

## Abstract

Phospholipids (PL) are widely used as aquafeed to enhance aquaculture production, particularly in crustaceans. The most common source of PL for aquaculture is lecithin, which is derived from both animals (e.g., eggs) and plants (e.g., soybeans). Including optimal levels of PL in crustacean diets enhances growth performance, survivability, antioxidant capacity, and lipid metabolism. PL is especially critical for early juveniles, as it supports osmoregulation by elevating Na+/K+-ATPase (NKA) activity. Furthermore, PL-enriched diets trigger energy metabolism, enhancing their physiological performance. In reproduction, PL provides energy for lipids mobilization and steroid transformation, improving the process of vitellogenesis in females. However, excessive PL can negatively affect the survival rate (SR), molting frequency (MF), condition factor (CF), and hepatosomatic index (HSI). This comprehensive review explores recent findings on how PL-enriched diets affect crustacean growth, metabolism, immunity, reproduction, gut microbiota, and osmoregulation. It also addresses gaps in our understanding of specific PL dietary needs for optimal crustacean health and productivity, offering evidence-based guidelines for effective PL supplementation in aquaculture.

## 1. Introduction

In aquaculture, fish feed is vital in supplying essential nutrients that promote the growth, survival, and overall health of cultured species. Fish meal and fish oil are excellent sources of protein and essential fatty acid (FA). Fish oil is preferred for polyunsaturated fatty acids (PUFA), such as eicosapentaenoic acid (C20:5, EPA) and docosahexaenoic acid (C20:6, DHA), whereas fish meal is crucial in meeting the demand for essential amino acids (EAA) for aquaculture [1, 2]. Feed formulations incorporating both marine and plant sources have been reported to effectively meet the nutritional requirements of fish and shrimp, both in terms of cost-effectiveness and nutritional balance [1, 3, 4].

Phospholipids (PL) from marine sources play a key role in modulating crustacean physiological functions, contributing to improved health. For instance, krill oil (KO)-derived marine PL has been reported to improve the gut immune barrier of female shrimp, reducing susceptibility to pathogenic bacteria and promoting growth [5]. This is consistent with the general benefits of PL-enriched diets, which support crustacean structural activity, metabolic, developmental, and physiological functions, compensating for limited endogenous PL synthesis [6, 7]. Marine organisms are a rich source of omega-3 (n-3) fatty acids (FAs), a key source of PL, particularly EPA, docosapentanoic acid (C22:5, DPA), and DHA [5].

Exogenous PLs are particularly beneficial for crustaceans during the highly stressful mature larval stages, a period of rapid physiological changes, where they potentially improve crustacean health and boost aquaculture productivity [8–10]. For instance, dietary PLs can enhance the immune response of cultured shrimp, making them resistant to bacterial and viral pathogens like *Vibrio alginolyticus* and *Litopenaeus vannamei* [11]. In addition, a study on *Portunus trituberculatus* showed supplementing the diet with 2.51% PL can increase progesterone (PROG) and 17 *β*-estradiol (E2) levels in the serum, while also improving cholesterol and triglyceride (TG) concentrations [12]. Furthermore, dietary PL can enhance the biosynthesis and accumulation of DHA and n-3 PUFA in hepatopancreas and improve the hepatopancreatic antioxidant capacity of crustaceans. However, the effectiveness of PL is affected by its dietary inclusion rates. For example, a rate of 0.99%–3.63% led to an increase in PL transfer protein (PLTP) activity, but a significant decrease in activity was observed when the inclusion rate ranged from 3.63% to 6.12% [13]. These results indicate that optimal PL inclusion boosts PL transfer protein activity, but excessive amounts reduce its effectiveness, highlighting the need for balanced PL levels in diets.

**Figure 1:**
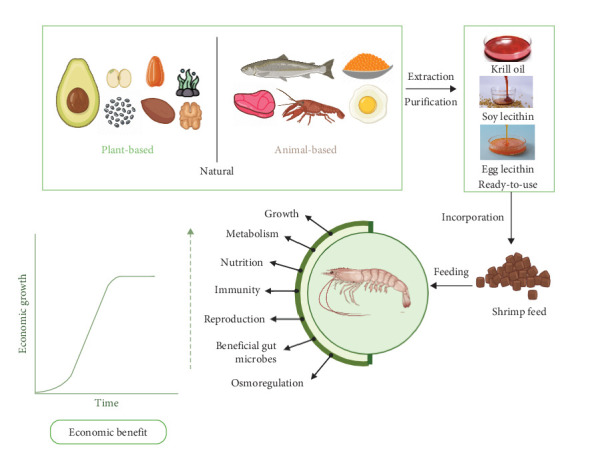
Function of dietary inclusion of PL in crustacean diet. This figure describes the process of shrimp feed production using natural ingredients. Purified krill oil, soy lecithin, and egg lecithin are extracted and incorporated into crustacean feed. This feed supports shrimp growth, metabolism, nutrition, immunity, reproduction, and overall health, leading to economic benefits through increased shrimp production. This figure was generated using Biorender.com based on published literature [15] (Agreement number: KS277QH1B8).

Moreover, PL is a source of important nutrients, including choline and essential fatty acids (EFAs), both of which are required for the growth and maintenance of crustaceans [14]. Therefore, incorporating PL in crustacean feed provides essential nutrients for the maintenance of optimal physiological functions, including growth, metabolism, nutrition, immunity, reproduction, gut microbiota, and osmoregulation as shown in Figure 1. These indicators will henceforth be referred to as physiological functions.

This review summarizes the findings from research and review articles over the past decade, emphasizing the role of PL in improving the physiological functions of culturable crustaceans, including shrimps, crabs, lobsters, crayfish, and prawns. Importantly, the review addresses a critical gap in the current limited understanding of the specific dietary PL requirements for optimal crustacean physiological functions. By providing evidence-based guidelines for PL supplementation, this review aims to enhance crustacean aquaculture productivity and health.

## 2. Methodology

Several electronic databases, including Scopus, PubMed, Web of Science, and Google Scholar, were searched for literature on the impact of PL-enriched diets on crustacean physiological functions. The search was limited to English-language scientific articles published between 2015 and 2024. Searches employed one-to-six-word phrases containing the keywords ‘PL' and ‘crustacean' along with over 100 additional keywords and search phrases (Table 1). Articles were selected based on several criteria, including coverage of one or more crustacean physiological functions, publication type (original research or review article), and peer-review status.

**Figure 2:**
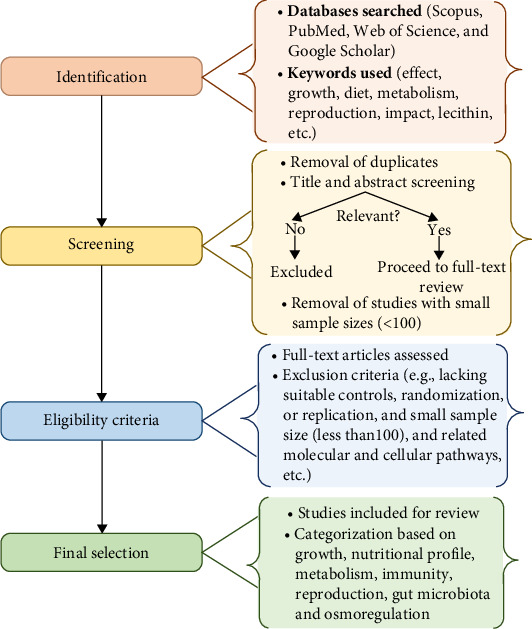
Flowchart of research methodology.

Subsequently, the quality of the selected articles was assessed based on standard criteria, including the clarity of study objectives, appropriateness of methodology, robustness of data analysis, and effectiveness of the findings. Following the initial synthesis of the findings, a careful cross-referencing and validation process was employed to find parallels and differences among studies, increasing the credibility and coherence of the results. Studies with flawed designs (no controls, randomization, or replication, and sample size <100) or poor data quality (incomplete, ambiguous, and inconsistent) were excluded to minimize bias and ensure reproducible evidence (Figure 2). A sample size of over 100 was used in this study to improve the reliability and generalizability of the synthesis. Small sample sizes are more susceptible to variability, bias, and lower external validity; hence, this criterion was chosen to select papers with strong statistical power and less sampling error. Previous systematic reviews have used comparable criteria to ensure that findings derived from the gathered information are both significant and repeatable.

## 3. Sources of PL in Crustacean Diet

Crustaceans can synthesize small amounts of PL, which is inadequate to meet their nutritional demands. Therefore, they rely on dietary PL from external sources to support physiological function [16]. Specifically, an estimated 10.31% PL in early juvenile diets inclusion is recommended for optimal growth, metabolism, immune response, and survival [17]. The potential of PL-enriched diets to exert a positive impact on crustacean's physiological functions has been reported [17–26]. This effect primarily depends on the crustacean species, PL sources, and inclusion rates. For instance, incorporating 1% soy lecithin (SL) in the *Scylla paramamosain* diet resulted in elevated levels of malondialdehyde (MDA), an immune parameter in crustaceans. However, the MDA levels were depressed when 4% SL was incorporated into *L. vannamei* diet [5, 22]. In other studies, 2% SL showed a positive impact on the survival rates (SRs) of *Penaeus monodon*, whereas similar inclusion rates resulted in lower SR in *S. paramamosain* [18, 19].

Crustacean aquaculture utilizes PL from both animal and plant sources, with lecithin being the most common [27]. SL is the most readily available plant-based PL, while egg yolk lecithin (EL) is a widely accepted animal-derived source. To further improve the availability of PL, KO, containing 30%–65% PL, has been increasingly explored as a major source [28]. Therefore, evidence suggests that the nutritional composition of PL-enriched diets is significantly influenced by their origin and source. For instance,SL, a plant-based lecithin, typically contains a lower amount of balanced FA profile compared to EL and KO, an animal-based lecithin (Table 2) [38].

### 3.1. Lecithin

Plant-based lecithin can be extracted from sources, such as soybean, sunflower, rapeseed, walnut, chia seed, camelina seed, and corn, whereas animal-based lecithin is sourced from egg yolk, krill, fish roe, and fish products, among others (Table 2). Among these, soy lecithin (SL), EL, and KO are the most utilized sources of PL in crustacean diets, each possessing distinct FA profiles [39]. Despite the availability of animal-derived lecithin, plant-derived lecithin, particularly SL, is generally favored in crustacean diets due to its greater availability [38]. This section will provide a detailed discussion of these major sources of PL, which are commonly used in aquaculture.

#### 3.1.1. SL

One of the most extensively used sources of PL in crustacean culture is soy lecithin (SL), a plant-based source containing 45.79% to 46.55% (wet weight basis) [40, 41]. Table 2 presents the general PL composition of soy lecithin, including phosphatidylcholine (PC), phosphatidylethanolamine (PE), inositol phosphatides (IPs)/phosphatidylinositol, and phosphatidic acid (PA). However, the specific composition can vary significantly depending on factors, such as the geographical location of the soybean, extraction methods, and quantification technique (Table 2). This structural organization of SL dictates its functionality, making it essential for maintaining the integrity of the biological membrane formation of cells and organelles. Consequently, this role supports energy production, particularly during the early developmental stages of aquatic organisms (Table 2).

The addition of SL to crustacean feed not only enhances its nutritional value but also acts as an emulsifying agent, potentially improving quality. The presence of PL helps to form a protective layer around the feed enhancing the stability and digestibility while mitigating leaching issues associated with water-soluble vitamins [39]. Moreover, SL provides EFAs and nutrients crucial for regulating physiological functions in aquatic organisms, especially during early juvenile stages. It is also a significant source of unsaturated fatty acids, n-3 and n-6, which are critical for optimal growth and health [42]. For instance, in numerous feeding trials on crustaceans, higher inclusion rates of PL-enriched feed have consistently demonstrated a positive impact on growth parameters, including weight gain (WG) and specific growth rate (SGR) [5, 11, 13, 18, 19, 43–47]. Notably, studies on *S. paramamosain* and *P. trituberculatus* crab, revealed that 4% SL inclusion resulted in the highest WG and SGR [19, 43]. Similarly, in *L. vannamei* shrimp, a 4% SL supplementation led to a low feed conversion ratio (FCR), leading to a high growth rate (6.71% SGR) [11].

The use of SL as the main source of PL has been shown to increase postlarvae tissue n-3 and n-6 FA in *P. trituberculatus*, indicating better lipid absorption [48]. This improvement is attributed to the conversion of PC to betaine, a known enhancer of lipid metabolism and energy production in crustaceans [49]. Similarly, studies on *Marsupenaeus japonicus* postlarvae showed increased HUFA absorption due to dietary SL. Betaine also enhances deoxyribonucleic acid (DNA) methylation by modulating the expression of immune-related genes, leading to an improved immune function mechanism in crustaceans [50]. This was evident in *L. vannamei*, which exhibited increased expression of immune-related genes, specifically toll-like receptor messenger ribonucleic acid (mRNA), immune deficiency (IMD) mRNA, and lysozyme (LZM) mRNA, with a 4% SL inclusion level [11].

Beyond its role in immune function and lipid absorption, SL also plays a vital role in the reproductive system of crustaceans by transferring lipids from the hepatopancreas to the ovary [20]. Several studies found that high inclusion rates of SL in crustacean diets can result in improved ovarian morphological structure, higher gonadosomatic index (GSI), and ovarian gene expression [20]. For instance, an inclusion rate of 6% SL resulted in positive GSI and provided a more ordered ovarian developmental structure in crayfish (*P. clarkii*) [44]. Likewise, higher SL has been associated with the enhancement of reproduction-related gene expressions, such as vitellogenin (VTG/VG) and fatty acid-binding proteins (FABP) gene, as shown in *L. vannamei* and *P. trituberculatus* [12, 13, 25]. As consistently shown in feeding trials, SL as a source of PL proves beneficial to crustaceans across various aspects of their physiological functions, contributing to improved health and aquaculture productivity.

#### 3.1.2. EL

Egg yolk is one of the most common sources of animal-based lecithin when considering yield and acceptability [51]. Several studies have investigated the significance of EL in different physiological attributes of crustaceans [17–19]. During the early stages of life, crustaceans depend on exogenous sources of PL for development, with their requirement increasing in the juvenile stages [52]. Compared with SL, EL presents a highly enriched and balanced FA profile with unique PL contents (ranging from 10%–54.3%) [41]. This superior FA profile of EL provides a greater amount of n-3 and n-6 FA, which are particularly advantageous during developmental stages. Additionally, n-3 and n-6 FA play a crucial role in cellular functions, immune modulation, and other relevant phenomena contributing to improved growth, SRs, immunity, metabolic function, reproductive capabilities, and molting frequency (MF) [20, 53].

There has been much interest in the use of EL to improve productivity in crustacean aquaculture. This is because of its demonstrated ability to enhance both growth and immune functions in crustaceans. For instance, studies have shown that including EL at a rate of 3% in the diets of *S. paramamosain* and *Cherax quadricarinatus* can improve their growth and immune indicators. These beneficial effects of EL were attributable to its ability to increase the activity of enzyme superoxide dismutase (SOD) [53]. Furthermore, EL exhibits antioxidant properties due to the presence of PL that are attached to the side chains of amino groups, such as those found in choline and ethanolamine [33]. This interaction creates a synergistic effect, ultimately resulting in antioxidant activity [23]. Additionally, investigations have indicated a correlation between elevated PL levels in cholesterol and accelerated MF in crustaceans. This is likely due to the high usage of cholesterol, which stimulates the secretion of ecdysteroid hormone that in turn binds to the ecdysone receptor (EcR) gene, regulating the molting process [16, 54]. Notably, a study on early juvenile green mud crabs (*S. paramamosain*) demonstrated a positive correlation between high EL inclusion and increased MF [55]. However, excessive EL inclusion can negatively impact crustaceans as shown by the lower growth rate of crabs (*P. trituberculatus*) fed on diets containing 4% EL. This suppressed growth of the crab was attributed to the replacement of triglyceride by PL leading to lower energy content [10, 13]. In summary, EL benefits crustaceans by providing EFAs and energy, thereby enhancing their bodily functions.

#### 3.1.3. KO

KO, particularly from Antarctic krill (*Euphausia superba*) a species rich in chitin, has recently gained popularity as a source of PL in crustacean feed due to its high lipid content [56]. KO contains around 51.7% lipids, with PL constituting a significant portion, ranging from 30% to 65% [34]. Notably, PC is the most abundant PL in KO, providing a potent combination of n-3 FA and choline, which plays a crucial role in the osmoregulation of crustaceans [57]. Moreover, KO is also a minor source of antioxidant, antibacterial, and immunomodulatory compounds, including vitamins, minerals, astaxanthin, and flavonoids [28, 58, 59].

Notably, KO contains elevated levels of n-3 PUFA like EPA and DHA, which contribute to various physiological processes in crustaceans, such as growth, immunity, reproduction, and metabolism. Owing to its rich lipid profile, KO exhibits high levels of n-3 PUFA, making it a potent growth promoter for crustaceans [45]. Other reports suggest higher bioavailability of n-3 PUFA from KO compared with SL or EL, translating to greater physiological benefits [5]. For instance, a study on Chinese mitten crab (*Eriocheir sinensis*) demonstrated superior growth performance with a 3% KO diet compared with diets containing the same ratio of SL or EL [45]. Similar positive effects were observed in Pacific white shrimp (*L. vannamei*), with improved survival and body weight when fed with KO compared to SL or EL. Moreover, KO may also contribute to enhanced immune response in crustaceans due to the presence of antioxidant nutrients like astaxanthin [60], though astaxanthin content varies widely between krill species, ranging from 40 to 5000 mg/kg [61].

A study by [21] on *Penaeus vannamei* and *E. sinensis* demonstrated increased expression of key immune parameters like glutathione peroxidase (GSH-Px/G-Px), prophenoloxidase (proPO), catalase (CAT), SOD, proPO activating enzyme, serine protease, beta-glucan binding protein, and hemocyanin gene activity with higher dietary inclusion of KO. Another group of researchers also investigated the role of KO in the deposition of yolk granules in female crustaceans. The findings suggest that KO can facilitate the mobilization of cholesterol from the hepatopancreas to the ovary, where it is converted into steroids. As a result, the ovaries acquire higher GSI and TG content, improved ovarian morphology, and ultimately, enhanced reproductive capacity [24]. To illustrate, a 70-day feeding trial on a prereproductive female *E. sinensis* showed that 2.5% KO resulted in better GSI, FA composition, and ovarian morphology. Compared to diets containing 2.5% SL and 2.5% El, the KO diet also upregulated the expression of ovarian genes, including vitellogenin receptor (VGR) mRNA and VG [23]. Consistent with this, a study investigating *C. quadricarinatus* found that a 2% KO inclusion rate yielded the highest GSI and the largest mature oocytes, suggesting comparable positive effects [20].

The impacts of KO diets extend beyond affecting crustaceans' physiological processes, as they also contribute to reducing gut microbes like Proteobacteria. This bactericidal effect of KO is attributed to the high levels of gut-derived endotoxin, such as lipopolysaccharide (LPS) that helps in alleviating injuries associated with these bacteria [62]. A recent study on *L. vannamei* showed that diets containing 4% KO resulted in lower populations of *Proteobacteria*, *Photobacterium*, and *Vibrio* compared to diets with 4% SL and 4% EL [5]. Therefore, incorporating KO in crustacean aquaculture can optimize health and developmental performance ensuring a more sustainable culture. While these benefits are clear, ongoing studies continue to evaluate the specific mechanism and extent of the positive impact of KO as a growth promoter and immunity enhancer [63].

Future research should focus on identifying novel, sustainable sources of PL, such as algae derivatives or microbial biomass. These alternative sources have ecological benefits and demonstrate potential for building a reliable and scalable supply chain for aquafeed production. Furthermore, the development of feed formulations that combine PL with other bioactive nutrients may reveal synergistic advantages for growth, immunity, and stress tolerance in aquaculture species. Investigating these interactions could improve the efficiency and cost-effectiveness of aquafeeds in the future.

### 3.2. Advantages and Disadvantages of Different PL Sources

SL is the most economically viable and widely available PL source for large-scale crustacean feed production, despite concerns over genetically modified organisms (GMOs) and lower purity [64, 65]. EL offers high-quality PL but is limited by high extraction costs and inconsistent supply [33, 38]. Krill oil lecithin (KOL) provides higher bioavailability and omega-3 but faces sustainability challenges due to limited harvests and ecological concerns [28, 66]. Among these sources, SL remains more feasible for long-term application in aquaculture systems.  Table 3 presents a comparative assessment of major PL sources, naming SL, EL, and KOL according to their nutritional properties, economic feasibility, and sustainability within crustacean aquaculture.

## 4. FA Profile of PL

FAs play a vital role in the biochemical and physiological functions of aquatic organisms by providing energy. They are particularly important for producing and maintaining permeability of cell membranes [67]. Different FAs, such as EPA and DHA, have unique features that significantly contribute to crustacean growth. EPA, a known precursor of eicosanoids, serves as an energy source in crustaceans, while DHA helps in maintaining membrane structures and functions [68]. Since crustaceans cannot synthesize all the FAs for optimal function, they must obtain some from external sources [69]. Notably, common dietary PL sources like SL, EL, and KO also provide the FAs required for better nutritional quality in crustaceans. The nutritional value of these sources is determined by their specific lipid composition and FA profile (Table 4). Therefore, supplementing crustacean diets with FA can maximize outcomes related to physiological functions [70].

The FA composition of SL, EL, and KO has been shown to significantly vary from one another. Specifically, SL primarily consists of linoleic acid (C18:2 n-6) followed by palmitic acid (C16:0) [71]. These two FAs play distinct roles in cellular functions, including maintaining cell integrity and acting as energy sources [72]. In contrast, EL contains higher levels of oleic acid, making it a valuable source of monounsaturated fatty acid (MUFA) for crustaceans. Functionally, oleic acid is known to improve feed efficiency (FE), cell signaling, and modulation of FA profile in aquatic organisms [73]. The FA profile of KO, a source of n-3 FA, exhibits a unique variation. In particular, it is characterized by the predominance of long-chain polyunsaturated fatty acid (Lc-PUFA), EPA, and DHA, which are associated with various health benefits, supporting growth and immunity in crustaceans (Table 4).

## 5. PL in Crustacean Growth

### 5.1. Effects of PL on Crustacean Growth

The global seafood market is projected to reach $139 billion by 2027, up from $113 billion in 2020 [74]. Crustaceans, notably shrimp and prawns, account for ~20% of global consumption [75]. Therefore, optimizing growth and survival during the rearing phase (culture period) is critical for advancing crustacean aquaculture [76]. However, crustaceans cannot fully synthesize all essential nutrients, constraining their production [16, 77]. Thus, they require supplementation for a balanced diet during their early life stages, particularly during the culture period.

PLs are among the most essential dietary components of the crustacean diet, playing a unique role in enhancing various physiological processes like growth and development [78]. Dietary PL, such as lecithin and KO, are important sources of essential nutrients, including inositol, choline, vitamins, phosphorous, EFA, and energy, which are key requirements in many physiological functions [79]. These components contribute to vital cell functions like the integrity of cell membranes, and boosting metabolic functions like digestion, absorption, and consumption of nutrients, lipids in particular [79]. Additionally, dietary PL, particularly from marine sources are rich in EPA and DHA, which act as growth promoters by fulfilling the high demands of rapid tissue formation [80]. Dayal et al. [18] demonstrated that a high percentage of PL sources, such as SL, results in improved lipid accumulation and energy availability, thereby boosting the circulation and transfer of lipids from the hepatopancreas to hemolymph and other tissues, which cumulatively enhances crustacean growth.

In a mitochondrial study on *P. trituberculatus*, the mitochondrial electron transport chain complex activity assay revealed higher mitochondrial complex II activity and mitochondrial membrane potentials in the hepatopancreas of the KO-fed crab [81]. Moreover, the benefits of PL extend to the improvement of wider growth parameters of crustaceans, including WG, final body weight (FBW), SR, SGR, MF, and condition factor (CF) (Table 5). However, some studies have observed potential negative effects on certain growth parameters with high PL inclusion. The following sections in this review explore these findings in detail, considering parameters like SGR, WG, FCR, SR, CF, FBW MF, FE, protein efficiency ratio (PER), hepatosomatic index (HSI), and relative growth rate (RGR) (Table 5).

There is growing evidence that PL-enriched diets have significant potential to enhance various growth parameters in crustaceans, particularly FBW, HSI, WG, RGR, SGR, and PER. Among the summarized data, FBW and WG exhibited the most consistent positive responses across multiple studies (10). Positive effects on SGR, SR, and MF were also observed in several studies (four to eight). However, parameters like PER, FCR, and RGR showed limited evidence of improvement, with only one to three studies reporting the gains. It is noteworthy that some parameters, including FCR, SR, and MF can be negatively or neutrally affected by PL inclusion depending on the specific inclusion rate and crustacean species (Table 5).

The impact of PL inclusion rates ranging from 0.99% to 6.12% was investigated on the growth of several crustacean species, including *P. trituberculatus*, *S. paramamosain*, *E. sinensis*, *L. vannamei*, *P. clarkii*, and *P. monodon* [5, 11, 13, 18, 19, 43–47]. In a study by Sun et al. [43], significant increases in FBW and MF for *P. trituberculatus* were found, rising from 3.69 to 44.48 g and 2.76% to 3.29%, respectively. However, another study on *P. trituberculatus* showed minimal improvement in FCR, with the lowest value (1.28) observed at a 4% inclusion rate of SL [43]. Correspondingly, a higher SR of 92.86% was observed in *S. paramamosain* due to 3% EL inclusion but lower gains of 86.90% at an SL inclusion rate of 2% in the same species were observed [43, 53]. From these studies, *S. paramamosain* achieved the highest WG (3267.41%) at a 3% PL inclusion, while *E. sinensis* (3.59% SGR) and *P. trituberculatus* (4.03% HSI) benefited most from 2% to 3% PL inclusion [19, 45, 47]. Collectively, these studies identified optimal PL inclusion rates for maximizing WG, SGR, and HSI [19, 46].

### 5.2. Mechanism of PL Effects on Crustacean Growth

**Figure 3:**
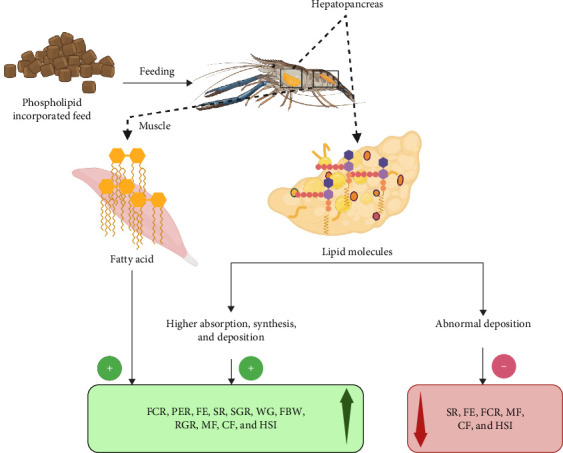
Role of PL on the growth of crustaceans. The figure depicts the effects of phospholipid supplementation on crustacean growth. PL plays a key role in energy storage and metabolism which are essential for growth and development in crustaceans. CF, condition factor; FBW, final body weight; FCR, feed conversion ratio; FE, feed efficiency; HSI, hepatosomatic index; MF, molting frequency; PER, protein efficiency ratio; RGR, relative growth rate; SGR, specific growth rate; SR, survival rate; WG, weight gain. This figure was generated using Biorender.com. To create the picture, the idea from [82] was followed. (Agreement number: UN277M10T7).

The optimized inclusion levels of PL in crustacean diets result in significantly improved growth performance and SRs across various species (Table 5).  Figure 3 illustrates the role of PL on crustacean growth parameters, demonstrating that PL enrichment primarily positively impacts SGR, WG, FBW, and RGR. The increase in these parameters due to dietary PL (EL and KO) is attributed to the elevated levels of n-3 FA, particularly EPA and DHA. Notably, EPA and DHA readily get absorbed and utilized by crustaceans [58], unlike linolenic (n-3) and linoleic acids (n-6 Lc-PUFA), which cannot easily be converted into EFA that is crucial for growth and molting [80]. Moreover, terrestrial PL sources like SL, enhance the growth conditions in crustaceans by increasing the surface area of lipid droplets in the aquafeed, resulting in higher digestibility of feed [83]. Positive impacts of PL diet on crustacean MF have been reported in many crustaceans, such as *E. sinensis* and *P. trituberculatus* (Table 5).

Aside from these growth parameters, PL-enriched diets can also improve SR, which is the number of individuals surviving the production [84]. The increased SR with PL inclusion was potentially linked to enhanced antioxidant activity in crustaceans, reflecting the complex interaction between PL-enriched feed and antioxidants present in crustaceans [11]. In another study, feeding trials on *P. trituberculatus* reported higher levels of HSI due to PL-enriched feeds [13, 85]. The hepatopancreas, a crustacean organ for the storage of organic and inorganic compounds, is evaluated using HSI to assess relative energy reserves [86]. As illustrated in Figure 3, increased HSI with PL inclusion occurs because of higher lipid absorption, synthesis, and greater hepatic lipid deposition [23].

Additional important growth parameters considered in feeding trials to assess the impact of PL-enriched diets include FCR, PER, and FE. Positive impacts of these parameters have been recorded in various crustacean species, often depending on the specific inclusion rate [11, 13, 18, 43, 85]. FCR, an indicator of FE, refers to the amount of feed needed to grow 1 kg (live weight) of aquatic organisms. Thus, lower FCR values signify better feed utilization, allowing organisms to grow in a less polluted waterbody [87]. Furthermore, PER, a measure of protein quality, is expressed as the ratio of body WG to protein consumed [88]. The positive impact of PL-enriched diets on these growth parameters can be associated with the increased availability of energy for growth. This is facilitated by enhanced lipid transportation and mobilization from the hepatopancreas to the hemolymph and other tissues [89].

However, studies have shown that at higher PL inclusion rates, some parameters like SR, MF, CF, and HSI may be reduced or experience no significant improvement [5, 19, 44, 45, 53]. For example, no significant effect was observed in SR and CF in *L. vannamei* fed with PL-enriched diets containing 0.05% to 0.1% cholesterol, yet feeding the same diets to *P. clarkii* resulted in decreased MF levels [5, 44]. Other studies investigating *S. paramamosain* and *E. sinensis* found decreases in MF and HSI at increasing PL inclusion rates, highlighting the importance of optimizing feed formulations with PL to ensure optimal growth performance [23, 53]. Collectively, these studies demonstrated that lower HSI values at higher PL inclusion rates in *E. sinensis* were attributed to the suppression of lipid uptake gene expression and a reduction in excess lipid synthesis. This aligns with observations from earlier studies on *P. trituberculatus* and *Larimichthys croceain*, which reported that excessive dietary lipid levels can lead to increased abnormal lipids deposition, resulting in metabolic disturbances and inflammation in organisms, ultimately inhibiting growth [48, 90].

## 6. PL in Lipid Metabolism

### 6.1. Effect of PL on Lipid Metabolism

Dietary PL supplies aquatic organisms with essential nutrients required for growth and development, particularly because of the inability of some species and juvenile crustaceans to biosynthesize it to meet their body requirements [16]. Primarily, the lipid-containing phosphorus plays a key role in enhancing lipid metabolism. PL emulsifies lipids, enhancing the bioavailability of crucial nutrients like EFA, phosphorous, choline, and inositol for vital biological functions [91]. Furthermore, dietary PL facilitates lipid transport from the gut epithelium to the hemolymph and various tissues, enabling the deposition of FA, cholesterol, and other lipophilic nutrients in crustaceans [92]. Dietary PL also serves as precursors for a wide range of biologically active lipid mediators critical to metabolism and physiological functions. These mediators, including eicosanoids, diacylglycerol (DAG), inositol phosphates (IP), and platelet-activating factors (PAFs), contribute to energy generation for normal physiological functions [93].

To further illustrate the influence of PL on lipid metabolism, studies employing molecular analysis of lipid uptake and synthesis genes in *E. sinensis*, such as fapt4, fatp6, fabp9, fabp10, fas, elovl6, and Δ9fad, support the role of PL-enriched diet in promoting lipid absorption and synthesis in the hepatopancreas, indicating improved overall metabolism [94]. Notably, a study on *S. paramamosain* revealed decreasing levels of srebp-1, fas, Δ6 fad, Δ9 fad, elovl4, and elovl6 with increasing PL inclusion rate where the highest fabp level was noted with 1% PL [82]. Indeed, extensive research has illuminated the metabolic benefits of PL-enriched diets for crustaceans. While most studies observed positive impacts of PL, the effect on specific metabolic parameters varies significantly with crustacean species, PL inclusion rate, and source. Therefore, the next section will examine the impact of PL on a range of key parameters, including TG, PL transfer protein (PLTP), low-density lipoprotein (LDL), high-density lipoprotein (HDL), total cholesterol content (T-CHO), very low-density lipoprotein (VLDL), phosphocholine cytidine transferase (PCT), carnitine palmitoyltransferase-1 (CPT-1), phospholipase A2 (PLA2), Acetyl-CoA carboxylase (ACC) and fatty acid synthase (FAS) as presented in Table 6.

Table 6 summarizes the findings from 10 feeding trials investigating the impacts of PL-enriched diets on crustacean metabolism. These studies explored the effects of varying inclusion rates (0% to 6.12%) of PL derived from various sources (SL, EL, and KO). Notably, PL-enriched diets demonstrated the potential to improve specific metabolic parameters. For example, nine studies reported a significant boost to T-CHO levels, while elevated TG levels were observed in seven studies. Furthermore, three studies indicated an increase in VLDL content, whereas increases in metabolic enzymes like PCT, PLA2, and CPT-1 were reported in one study. In contrast, higher PL inclusion rates did not consistently yield positive effects as demonstrated in two studies that reported a negative impact on TG, HDL, and LDL, and one study that observed lower levels of PLTP, FAS, and ACC.

The effects of PL enrichment on individual crustacean species, as detailed in Table 6, reveal substantial variations in lipid metabolism. For instance, *P. trituberculatus* exhibited a notable increase in hepatopancreas T-CHO, reaching a maximum of 13.20 μg/mg when fed a diet containing 2% SL [47]. Similarly, *S. paramamosain* showed elevated TG levels (5.89 mmol/gprot) with 4% EL inclusion, but conversely, a lower inclusion rate (0.5%) resulted in decreased TG (2.80 mmol/gprot) [22, 57]. Significant increases in HDL and VLDL were also reported in *E. sinensis* due to PL-enriched diets [23]. This study found an increase in HDL to a maximum of 4.2 mmol/L with a 2.5% SL diet, while VLDL peaked at 2 mmol/L with 2.5% KO. Moreover, *L. vannamei* showed increased LDL levels to a maximum of 1.1 mmol/L with 4% KO, and a minimum of 0.85 mmol/L at 0% KO inclusion [24]. The variation in the responses to PL enrichment can be attributed to differences in lipid metabolic enzymes across species and inclusion rates as illustrated in several studies. Increased CPT-1 activity was reported in *C. quadricarinatus* with 3% SL [26], a response that was mirrored in *P. trituberculatus* fed with 3.63% SL, where PCT and PLA2 increased to a maximum of 105.18 and 47.37 U/mgprot, respectively [13]. However, some studies reported negative and contradictory results. For instance, while 1% SL resulted in the highest TG levels (3.10 mg/dl) in *P. trituberculatus*, 2% SL yielded the lowest (1.84 mg/dl) [47]. These variations in effects must be considered when developing species-specific and context-dependent PL inclusion strategies for crustacean aquaculture.

### 6.2. Mechanism of PL Effects in Crustacean Lipid Metabolism

**Figure 4:**
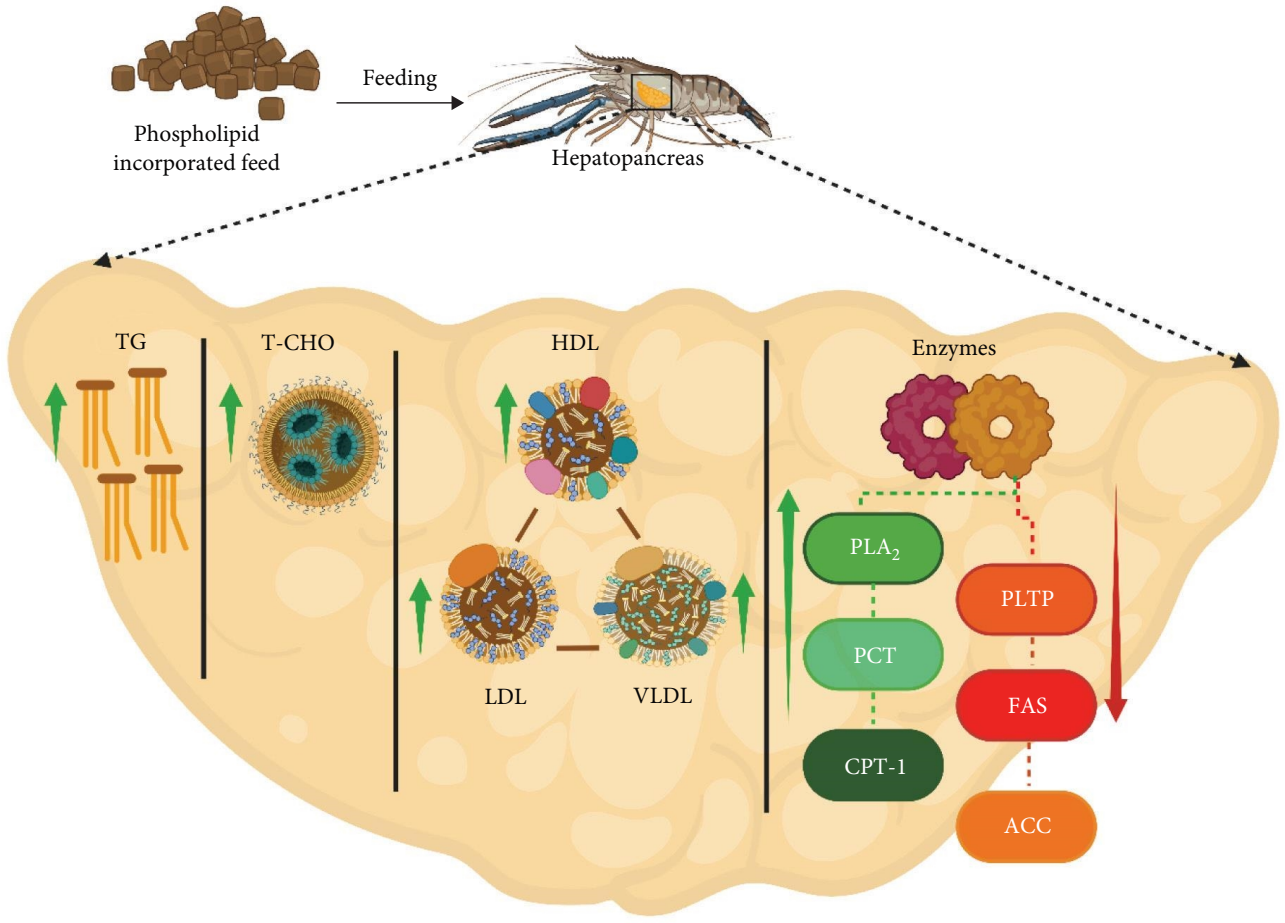
Impact of dietary phospholipids in crustacean metabolism. The figure depicts the effects of phospholipid-enriched feed on lipid metabolism in crustaceans. Phospholipid enhances the absorption, synthesis, and deposition of fatty acids and lipid molecules in the muscle and hepatopancreas which leads to improved health parameters. ACC, acetyl-coa carboxylase; CPT-1, carnitine palmitoyltransferase-1; FAS, fatty acid synthase; HDL, high density lipoprotein; LDL, low density lipoprotein; PCT, phosphocholine cytidine transferase; PL, phospholipid; PLA2, phospholipase A2; PLTP, phospholipid transfer protein; T-CHO, total cholesterol content; TG, triglyceride; VLDL, very low density lipoprotein. The figure was generated using Biorender.com from the concepts of [97] (Agreement number: IB277QKHNX).

Dietary PLs are essential for various cellular functions, including signaling, transportation, and regulation of membrane-bound enzyme activity [96]. In crustaceans, the hepatopancreas serves as the central regulatory organ for PL metabolism and storage (Figure 4), which dictates how dietary PL supplementation influences lipid distribution and metabolism [98]. In particular, supplementation with PL induces an increase in hepatopancreas lipids and a decrease in muscle lipids, implying that PLs aid in lipid deposition in the hepatopancreas while enhancing lipid catabolism in the muscle. Therefore, PL supplementation in crustacean diets can significantly affect various lipid metabolic factors, including HDL, LDL, VLDL, TG, T-CHO, and lipid metabolism enzymes (ACC, CPT-1, PCT, PLA-2, PLTP, FAS) (Table 6).

Lipoproteins (HDL, LDL, and VLDL) function as cholesterol transporters in the body, of which HDL exhibits a high protein-to-lipid ratio and is considered beneficial due to its inverse association with the risk of heart diseases [99]. Conversely, LDL, which primarily transports cholesterol, is associated with cardiovascular risks. VLDL plays a vital role in transporting TG from the hepatopancreas to the perihepatic tissues. This function is supported by supplemented PL, which maintains proper VLDL assembly and secretion into the serum [23, 45]. Similarly, VLDL is also regarded as a “bad” cholesterol due to its association with cardiovascular disease risks [100]. An increased or decreased HDL/LDL ratio reflects whether cholesterol in the serum is taken up by the hepatopancreas for metabolic activities or released from the hepatopancreas to peripheral tissues [95].

To illustrate the diverse effects of PL supplementation on crustacean lipid metabolism, feeding trials on *E. sinensis* demonstrated an increase in HDL, with the highest HDL (4.2 mmol/L) in the hepatopancreas reported at 2.5% SL inclusion rate. Conversely, a rapid decrease was observed in *S. paramamosain* fed a 1.5% SL diet, yielding the lowest HDL level (0.01 mmol/L) [22, 23]. Additionally, the highest VLDL level was reported in *E. sinensis* with 5% SL supplementation [94]. Despite these findings with SL, a recent study by [24] suggested that EL and KO supplementation may be more effective in promoting TG transport from the hepatopancreas to the ovary, potentially due to enhanced lipoprotein formation.

To further elucidate the mechanism of PL in crustacean lipid metabolism, dietary PL has been proposed to promote the absorption of cholesterol, TG, and n-3 FA from the gastrointestinal (GI) tract to the hepatopancreas. Subsequently, the absorbed lipids are delivered to various tissues through the hemolymph, resulting in elevated TG levels [6, 101]. Recently, [57] observed the highest TG accumulation (5.89 mmol/gprot) in *S. paramamosain* fed with an EL-supplemented diet. This increment was attributable to improved lipid emulsification by PL, facilitating the transportation process from the hepatopancreas to the hemolymph. Additionally, enhanced emulsification may have promoted lipid deposition and energy utilization within crustacean tissues [47].

Figure 4 summarizes the central role of crustacean hepatopancreas in bridging between the synthesis and secretion of digestive enzymes, lipid metabolism, nutrient absorption, and transportation. For instance, FAS, the primary limiting enzyme for lipid synthesis, predominantly found in the hepatopancreas and ovaries [102], exhibits a dose-dependent response to PL inclusion. Within an optimal range, PL supplementation enhances FAS activity, while excessive PL can lead to inhibition [13]. Similarly, PCT catalyzes a key regulated step in the cytidine diphosphate (CDP)-choline pathway, controlling the rate of PC and PC-related lipid production [103]. Furthermore, CPT-1, a mitochondrial membrane protein, regulates the rate-limiting step in FA oxidation, facilitating the transport of long-chain FA from the cytoplasm into mitochondria for oxidation [104]. Additionally, PLA2 enzymes play a regulatory role in the eicosanoid pathway by releasing free arachidonic acid from membrane PL, whereas ACC plays a crucial role in both the synthesis and oxidation of FAs, providing energy for cellular functions [105, 106]. Therefore, considering these enzymatic roles within the context of the central function of hepatopancreas, it becomes evident that lipid deposition in crustaceans is ultimately a product of the delicate balance between internal lipid synthesis and catabolism, both of which are significantly modulated by dietary PL [107].

## 7. PL on the Nutritional Profile of Crustaceans

### 7.1. Effect of PL on the Nutritional Profile of Crustaceans

Lipids are essential constituents of crustacean diets, playing multiple functions, including a source of energy source, supply of EFA, carrier for fat-soluble vitamins, formation of structural components for biomembranes, and serving as precursors for vital biological compounds like hormones and enzymes [108]. Notably, crustaceans cannot synthesize n-3 and omega-6 FA, and therefore they must be acquired from the diet. Specifically, alpha-linolenic acid (ALA) and linoleic acid (LA) serve as precursors for n-3 and n-6 PUFA, respectively [109].

Given the importance of lipids, numerous studies have investigated the effects of PL-enriched diets on crustacean nutrition (Table 7). These studies reveal significant changes in various FA components, including saturated fatty acid (SFA), MUFA, PUFA, EPA, and DHA in response to varying PL inclusion rates. However, some nutritional parameters were observed to decrease with PL-enriched diets. Therefore, this review aims to elucidate the effects of (saturated fatty acid) SFA, MUFA, n-3 PUFA, n-6 PUFA, Lc-PUFA, EPA, and DHA on crustacean nutrition. From these studies, we can observe widespread increases in DHA levels with increasing PL inclusion rates. However, PUFA levels showed a contrasting pattern, with decreases appearing in five studies. Furthermore, SFA levels exhibited mixed results, increasing in six studies and decreasing in two. Similarly, EPA levels increased in five but decreased in three. MUFA levels displayed the most inconsistent responses, with four studies showing increases and four showing decreases (Table 7).

The influence of PL inclusion on nutritional parameters demonstrates a species- and dose-dependent response. For instance, *E. sinensis*, *P. trituberculatus*, and *S. paramamosain* showed positive responses for most nutritional parameters at increasing PL-inclusion rates, except in MUFA and PUFA levels [19, 43, 45, 47, 53, 57]. For instance, *S. paramamosain* exhibited 46% SFA of total FA with a PL diet constituting a 4% level. This was significantly higher compared with the 31.71% SFA reported with a lower 4% PL inclusion rate for the same species [53]. Interestingly, *S. paramamosain* exhibited the highest PUFA level (21.04% of total FA) with 4% PL inclusion but a higher 6% PL inclusion level reversed this trend, resulting in decreased PUFA levels in *P. clarkii* (31.66%) [63, 97]. Finally, the DHA levels in *S. paramamosain* consistently increased with PL incorporation, while the EPA level decreased with a 3% inclusion rate and increased at a 2% PL-inclusion rate [53].

### 7.2. Mechanism of PL Effects on the Nutritional Profile of Crustaceans

**Figure 5:**
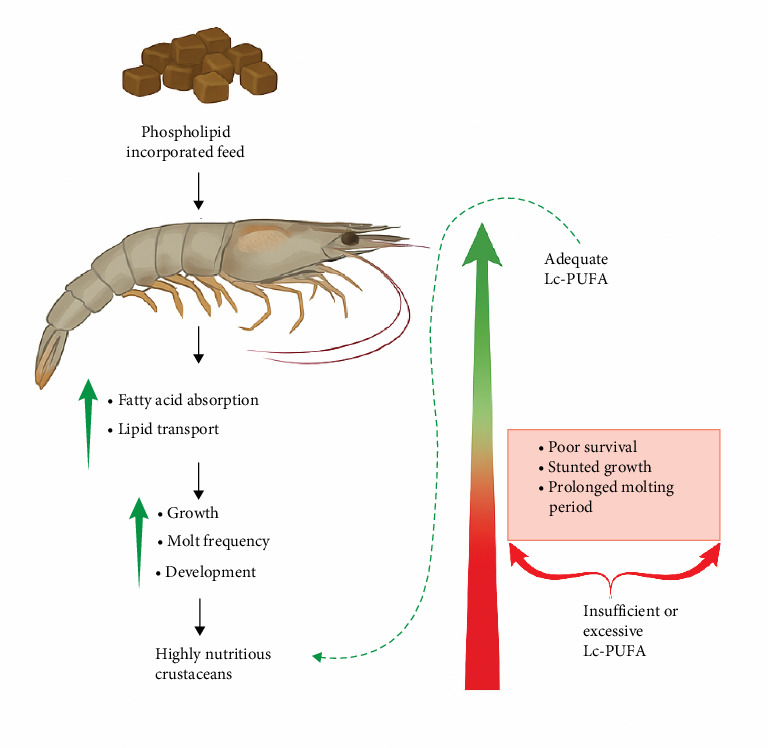
Functionality of dietary phospholipids in crustacean nutrition. The figure illustrates the effects of phospholipid-enriched feed on the fatty acid composition of crustacean muscle. It shows that feeding crustaceans with PL supplementation can increase the levels of beneficial fatty acids like MUFA, PUFA, and HUFA. HUFA, highly unsaturated fatty acid; MUFA, monounsaturated fatty acid; PL, phospholipid; PUFA, poly unsaturated fatty acid; SFA, saturated fatty acid. The figure was generated using Biorender.com (Agreement number: OA277QJR3Z).

Dietary inclusion of PL at optimal levels has demonstrated positive effects on various nutritional parameters in crustacean species (Figure 5) [110]. Crustaceans, like most marine fish, have a limited capacity to convert linolenic and linoleic acids to n-3 and n-6 Lc-PUFA, making dietary Lc-PUFA essential [80]. However, insufficient or excessive Lc-PUFA often results in a negative impact on crustaceans, including poor survival, stunted growth, and prolonged intermolt phases [111]. Thus, maintaining functional dietary EFA levels is critical for growth, development, and survival [112]. Dietary PL enhances Lc-PUFA absorption efficiency and could potentially improve lipid transport in swimming crabs [113].

DHA and EPA are critical for optimal crustacean growth, molting, and development. On the one hand, DHA is an essential constituent of biomembrane structure, particularly forming a key component of polar lipids in neural tissues (brain and eye) [80]. Consequently, there is an increased demand for DHA during periods of rapid growth to support developing tissues. On the other hand, EPA plays a significant role in the production of eicosanoids, bioactive regulatory compounds, and can contribute to DHA synthesis in some species with the necessary enzymes [114]. However, an imbalanced dietary DHA/EPA ratio can hinder neural development and immunity, potentially resulting in slowed growth or poor SRs of marine animal larvae. Moreover, high levels of EPA might also be detrimental to aquatic organisms [115]. Therefore, establishing optimal DHA/EPA ratios in diets is crucial for the healthy growth and development of crustaceans [80].

## 8. PL and Immunity

### 8.1. Effect of PL on the Immunity of Crustaceans

Crustaceans rely solely on an integrated innate immune system, combining cellular and humoral defenses [116]. Owing to this, their immunity is vulnerable to stressors like pathogens, environmental changes, physiological issues, and molting [117–120]. Molting, however, can enhance immunity by upregulating antimicrobial peptides, as demonstrated in recently molted mud crabs [120].

Dietary PLs have been investigated for their potential to modulate the crustacean immune system.  Table 8 summarizes 12 studies examining the effects of various sources of PL (SL, EL, and KO) at different inclusion rates (0%–6.12%) on various immune parameters across different crustacean species. Overall, the data suggests that dietary PL can enhance the immune system and the expression of immune-related genes in different crustaceans. However, the effects of PL supplementation are not uniform. While many studies report a positive correlation between moderate to high PL inclusion and increased levels of parameters, like SOD, GSH-Px/G-Px, ProPO, total-antioxidant capacity (T-AOC), and LZM [11, 13, 16, 17, 20–22, 43–45, 53], others observe decreased levels of specific immune parameters at high PL inclusion.

For instance, significantly higher SOD activity (an increase from 317.81 to 418.96 unit/mL) was observed in *L. vannamei* with a 4% PL inclusion [11]. Similarly, CAT activity was also observed to increase in *E. sinensis* with a 2.5% KO inclusion rate (7 U/mgprot) [23]. On the contrary, variable CAT responses were reported in *P. clarkii* with increasing SL inclusion where a 6% SL inclusion resulted in 1.18 U/mL while an SL-free diet produced a 2.08 U/mL CAT activity [44]. Furthermore, parameters like MDA exhibited species-specific responses, exhibiting more downregulation than upregulation. For example, MDA levels decreased in seven studies with higher PL diets but increased in three [5, 17, 20, 23, 43, 45, 53] (Table 8).

Moreover, a few other parameters, such as T-AOC, LZM, alkaline phosphatase (AKP), acid phosphatase (ACP), and IMD mRNA, displayed occasional decreases in some studies, likely influenced by factors like species, PL source, and inclusion rate [11, 44]. These studies showed that the level of GSH-Px, T-AOC, and GSH increased in *P. trituberculatus*, exhibiting 328.14 U/mgprot (4% SL), 4.38 U/mgprot due (4.95% SL) and 251.34 μmol/gprot (3.63% SL), respectively [13, 43]. Another immune parameter, LZM, showed a positive response (123.53 U/mL) in *P. clarkii* due to a 2% SL inclusion rate [44].

### 8.2. Mechanism of PL Effect Crustacean Immunity

**Figure 6:**
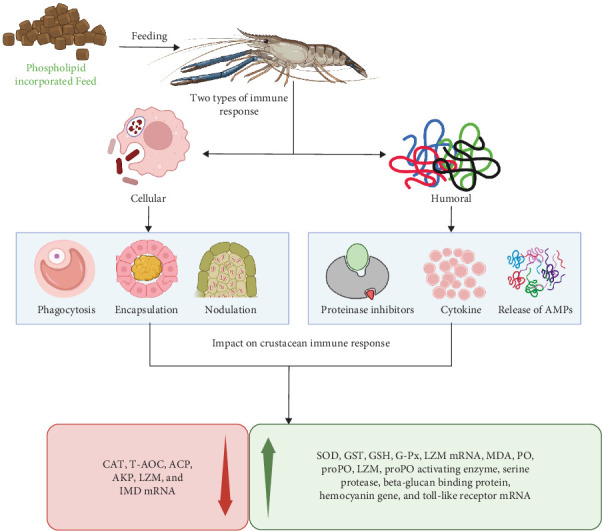
Mode of action of phospholipids in crustacean immunity. The figure describes how supplementation of phospholipid in the diet affects the immune response of crustaceans. It exhibits that feeding crustaceans with PL supplementation can stimulate both cellular and humoral immune responses, leading to increased phagocytosis, encapsulation, nodulation, proteinase inhibition, cytokine release, and the expression of various immune-related genes. This overall enhancement of the immune response can improve the health and survival of crustaceans. ACP, acid phosphatase; AKP, alkaline phosphatase; CAT, catalase; GSH, glutathione; GSH-Px, glutathione peroxidase; GST, glutathione S-transferase; IMD mRNA, immune deficiency messenger ribonucleic acid; LZM, lysozyme; MDA, malondialdehyde; SOD, superoxide dismutase; T-AOC, total antioxidant capacity. The figure was generated using Biorender.com from the idea of Adel and Dawood [121] (Agreement number: GZ277M0E8Y).

Figure 6 illustrates the diverse pathways through which dietary PL influence crustacean immunity, primarily by targeting humoral components. These components form the main line of defense against oxidative stress, with enzymes, such as SOD, CAT, T-AOC, GST, GSH, and GSH-Px playing key roles [122]. The enzymes remove hazardous toxins from the body and scavenge reactive oxygen species (ROS), protecting against oxidative stress and cell damage [123]. For instance, the ROS scavenging property of T-AOC, CAT, SOD, GSH, and GSH-Px acts in the first-line antioxidant defense mechanism. GST, on the other hand, modulates the immune response and mechanism and reduces cell damage caused by ROS [124]. Furthermore, GST activity can be used as a biomarker to assess exposure to organic pollutants in aquatic organisms [125]. Studies on crustacean species, including *C. quadricarinatus*, *Penaeus vannamei*, *S. paramamosain*, *E. sinensis*, *L. vannamei*, and *P. trituberculatus* have demonstrated the positive impacts of dietary PL supplementation on these six previously mentioned parameters [5, 11, 13, 20–23, 43–45, 53]. However, studies on *P. clarkii* exhibited a decrease in T-AOC content with increasing PL doses [44]. This variation in the antioxidant immune response of crustaceans due to a high PL diet is not well known but it can be possible due to the complex interaction between PL feeds and antioxidants [11]. It is also possible that the observed improvements in immune parameters might be due to the presence of high levels of linolenic acid and linoleic acid in the PL supplements [126].

MDA serves as another crucial immune-related antioxidant enzyme and biomarker in crustaceans which plays a role in free radical-induced lipid peroxidation, a process that can damage cells through oxidative stress [13, 127]. MDA levels fluctuate depending on the crustacean species and the amount of PL included in their diet. For instance, species like *P. trituberculatus*, *S. paramamosain*, *E. sinensis*, *L. vannamei*, and *C. quadricarinatus* showed decreased MDA levels with increasing PL intake, whereas others like *S. paramamosain*, *P. clarkii*, and *P. trituberculatus* exhibited the opposite trend [5, 17, 20, 22, 23, 43–45, 53]. Intriguingly, some studies on *P. trituberculatus* displayed both increased and decreased MDA levels, depending on the PL inclusion rate (6.12% vs. 4% SL, respectively) [13, 43]. Therefore, decreased MDA levels may indicate relief from oxidative damage, while increased levels could reflect lipid peroxidation and oxidative damage induced by high dietary lipid content [19, 128].

LZM, another key ubiquitous enzyme in crustacean immunity, lyses peptidoglycan cell walls of bacteria, particularly gram-positive ones, enhancing the nonspecific immune response [129]. PL-enriched diet can significantly increase LZM activity in crustaceans as demonstrated in *P. clarkii*, and *L. vannamei* fed with high PL doses [5, 44]. Furthermore, PL enriched diet also exerts a distinct effect on the expression of immune-related genes, including toll-like receptor mRNA, IMD mRNA, and LZM mRNA [11]. Variations in mRNA expression levels of proPO activating enzyme, serine protease, beta-glucan binding protein, and hemocyanin gene have also been reported in response to PL diets [21]. These effects are likely linked to higher levels of EPA, and DHA in the PL supplements, which are known to promote immune gene expression [130]. Additionally, the amino acid composition of PL-enriched feeds may contribute to enhanced immune gene expression like toll-like receptor mRNA, IMD mRNA, and LZM mRNA [131].

Moreover, ACP and AKP activities are also crucial parameters in crustacean immune defense mechanisms. ACP aids in digesting invading pathogens, while AKP facilitates detoxification, improved digestion, and nutrient absorption [132]. Both enzymes catalyze the hydrolysis of various phosphate groups, further improving the immune capacity of aquatic organisms [133, 134]. However, a study on *P. clarkii* fed with 6% SL reported lower activity levels of AKP and ACP [44], highlighting the need for further research to understand the specific factors influencing the impact of PL inclusion levels on different enzymes.

## 9. PL and Reproduction

### 9.1. Effect of PL Enriched Diet on the Reproduction of Crustaceans

Vitellogenesis, the lipoprotein-based yolk formation process, is crucial for ovarian maturation in crustaceans [135]. Cholesterol, a key component of lipoproteins is derived from dietary PL, particularly PC and PE [20]. Its level in the hemolymph decreases gradually following yolk maturation, suggesting its role in enhancing secondary vitellogenesis and ovarian development in female shrimp [16]. It is important to note that, environmental factors like salinity, temperature, and photoperiod also play significant roles in crustacean reproduction [136]. For example, studies on crab *Scylla olivacea* and shrimp *Neocaridina davidi* demonstrated how temperature and salinity variations impact ovarian development [137, 138]. Similarly, the effect of photoperiod variation on the reproductive performance of *Callinectes sapidus* has also been reported [139].

While feeding trials often show the positive influences of PL-enriched diets on female reproductive performance, the specific mechanisms of crustacean reproduction remain under investigation. To elucidate these effects and their relevance to sustainable crustacean farming, eight studies examining the impact of PL-enriched diets on crustacean reproduction are summarized in Table 9.

The studies presented in Table 9 demonstrate the role of various PL sources (SL, EL, and KO) in crustacean diets at inclusion levels ranging from 0% to 8%. Overall, PL incorporation led to improved female reproductive performance, as evidenced by higher GSI in five studies, increased ovarian quality in four studies, and elevated levels of VG/VTG gene expression and reproductive hormones (E2, PROG) in three studies. Additionally, MF levels and VGR gene expression also showed increasing levels in two and one studies, respectively. However, some parameters were negatively affected (decreased) on PL inclusion, suggesting species-specificity and dependance on PL source and inclusion rates. For instance, levels of GSI and some hormones (LH, E2) decreased in one study, while gonadotropin-inhibiting hormone (GIH) and molting inhibiting hormone (MIH) were lower in two studies (Table 9).

The studies revealed that high to moderate PL inclusion rates (2%–6%) resulted in improved reproductive parameters. GSI increased in *C. quadricarinatus*, *P. clarkii*, *L. vannamei*, *E. sinensis*, and *P. trituberculatus* at these levels [12, 13, 20, 23, 95]. Notably, *E. sinensis* displayed a significant increase (7%–9% GSI) with 2.5% KO supplementation [23]. Furthermore, high to moderate PL-inclusion rates improved ovarian histology, ovarian TG, (reproductive hormone concentration) RHC, FA composition of the ovary, and overall ovarian morphology in *C. quadricarinatus P. clarkii*, *L. vannamei*, and *E. sinensis* [20, 23, 95]. Moreover, genes associated with female crustacean reproduction, vitellogenesis (VGR, VTG/VG, and FABP), also responded positively to PL enrichment. *E. sinensis*, *L. vannamei*, and *P. trituberculatus* exhibited increased expression of these genes with 2.5%–6% dietary PL [23, 25, 95].

### 9.2. Mechanism of PL in Crustacean Reproductive Organ Development

**Figure 7:**
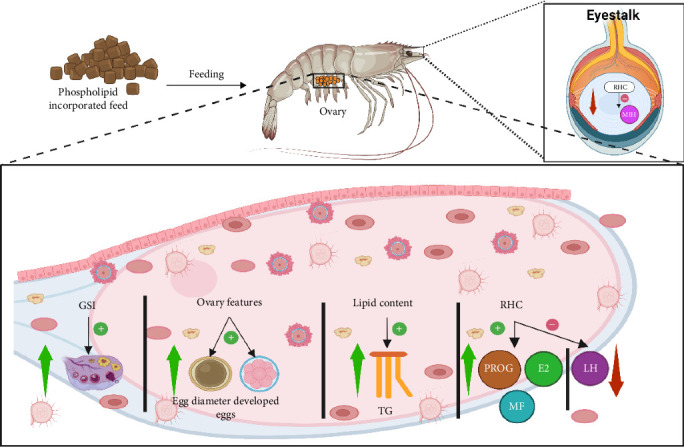
Impact of phospholipids on crustacean reproduction. E2, estradiol; GSI, gonadosomatic index; LH, luteinizing hormone; MF, methyl farnesoate; MIH, molting inhibiting hormone; PROG, progesterone; RHC, reproductive hormone concentration; TG, triglyceride. The figure was generated using Biorender.com (Agreement number: HX277LZLJ2).

The GSI, which refers to the ratio of the gonad weight to body weight, is a reliable parameter for estimating the reproductive condition of female crustaceans [140, 141].  Figure 7 depicts the impact of PL supplementation on crustacean reproduction. It shows that PL-enriched feed can enhance the GSI, improve ovarian features, enhance lipid content, and explicitly regulate reproductive hormones including PROG, E2, and luteinizing hormone (LH). Studies show increased GSI in *C. quadricarinatus*, *P. clarkii*, *L. vannamei*, *E. sinensis*, *L. vannamei*, and *P. trituberculatus* with 2%–6% PL inclusion rate [13, 20, 23, 24, 95]. From these studies, the increase in GSI can be attributed to the elevated EPA and DHA deposition in the hepatopancreas in the PL-fed crustaceans [20]. However, limitations of GSI as an indicator of reproductive cycle performance in crustaceans have been recognized, as it offers limited insights into internal ovarian changes, particularly maturation [142]. Therefore, to gain a comprehensive understanding of gonadal development in response to PL-enriched diets, researchers have employed additional histological and biochemical parameters, including ovarian histology, ovarian TG, RHC, ovarian FA composition, and ovarian morphology [20, 23–25, 95]. These studies consistently demonstrated a positive impact on all parameters. For example, 2% KO supplementation in *C. quadricarinatus* supported the production of larger mature oocytes and elevated ovarian TG levels (0.5 mmol/gprot) [20].

Regarding ovarian development and morphology, *P. clarkii* exhibited a more compact and advanced structure with 6% SL supplementation [95]. In *E. sinensis*, increasing PL level positively impacted FA composition, resulting in elevated total Lc-PUFA and n-3 FA, likely because of higher deposition of lipids in the ovarian muscle [23]. This is significant as lipids are crucial in yolk biosynthesis and oocyte development, thereby promoting gonadal development [16].

Furthermore, higher levels of RHC were observed in *C. quadricarinatus*, *E. chinensis*, and *P. trituberculatus*, while elevated Vg gene expression was reported in *E. chinensis*, *P. trituberculatus*, and *L. vannamei* with increased PL intake [12, 13, 23, 24, 57]. This upregulation was attributed to the neuroendocrine regulatory network governing crustacean reproduction [46]. E2, a key sex steroid, is reported to exert a physiological influence on female crustaceans reproductive performance [143]. E2, along with MF, acts as a precursor for VG synthesis in the liver and oocyte development [24, 144]. As presented in this review, dietary PL provides energy for lipid mobilization and conversion to steroids, ultimately increasing VG levels [23].

Notably, a cellular study on *L. vannamei* identified distinct metabolic pathways for glycerophospholipid, glycosylphosphatidylinositol (GPI)-anchor biosynthesis, alpha-linolenic acid, arachidonic acid, and glycerolipid for various PL-enriched feed. However, sphingolipid metabolism was observed exclusively in KO-fed shrimp, suggesting a higher energy providing capacity of KO during ovary development stages through enhanced lipid utilization [24].

## 10. PL and Gut Microbiome

### 10.1. PL on Gut Microbiome in Crustaceans

The gut microbiome plays a crucial role in crustacean health, influencing nutrient absorption, immune response, homeostatic balance, and overall productivity [145, 146]. Specific microbial communities within the crustacean gut serve as a first line of defense against pathogens and contribute to essential physiological functions [146–148]. These communities vary depending on rearing stage, diet, and health status, with Firmicutes, Proteobacteria, and Bacteroidetes being the dominant phyla [149–153]. For instance, the dominant phyla differ between *E. sinensis* (Proteobacteria and Tenericutes), *S. paramamosain* (Firmicutes, Proteobacteria, Bacteroidota, and Campilobacterota), and *Penaeus* sp. (Proteobacteria) [154–156].

Bioinformatics tools have been developed to predict variation in microbial diversity across samples [157]. One prominent tool, operational taxonomic units (OTUs) utilizes sequence data to define microbial communities across different taxonomic levels (phylum, class, order, family, genus, and species) [158]. OTUs are employed alpha diversity, which represents the average diversity of microbial species within a specific habitat. Commonly used alpha diversity indices are Simpson, Shannon, Abundance-based Coverage Estimator (ACE), and Chao1. The Simpson and Shannon indices measure species richness based on the degree of dominance by individual species. Conversely, the ACE index estimates the sample completeness or coverage, indicating how well a sampling effort captures the entire microbial population. Chao1 index requires abundance data from individual samples to determine species richness [159, 160].

PL-enriched diets can influence gut microbiome composition and diversity. However, these effects have received less attention compared to growth, reproduction, immunity, or nutrient metabolism. Feeding trials have investigated gut microbiome composition and diversity using parameters, such as richness index indicators (Shannon, Simpson, ACE, and Chao1) and dominant phyla. The impact of different PL sources (SL, EL, and KO) on crustacean gut microbiota is summarized in Table 10. Richness indices (Shannon, ACE, Chao1) increased with increasing dietary PL levels in three studies, whereas the Simpson index exhibited variable responses, showing increases in two studies and a decrease in one (Table 10). Commonly observed phyla included Proteobacteria, Firmicutes, Actinobacteria, Tenericutes, and Bacteroidetes, with prevalent genera, such as *Aeromonas*, *Vibrio*, *Candidatus*, *Bacilloplasma*, *Citrobacter*, *Flavobacterium*, and *Shewanella* [5, 26, 44].

The observed variations in gut microbiota depend on PL inclusion rate and specific crustacean species. For example, *C. quadricarinatus* exhibited a significant increase in the Shannon index (3.0) with 2% SL inclusion, while ACE and Chao1 indices reached their maximum values (~510) with 4% KO supplementation and minimum values (350) with 0% inclusion. Moreover, the highest Simpson index was recorded in *P. clarkii* (0.16) and *L. vannamei* (0.8) because of the 2% SL and 4% KO inclusion, respectively. Contrary, the Simpson index decreased in *C. quadricarinatus* (0.11) with 1% SL inclusion compared to 0.32 in PL-free diet. These findings suggest that PL can enhance gut microbiome richness and diversity in crustaceans, potentially contributing to improved health [161].

### 10.2. Mechanism of PL Enriched Diet on Crustacean Gut Microbiota

**Figure 8:**
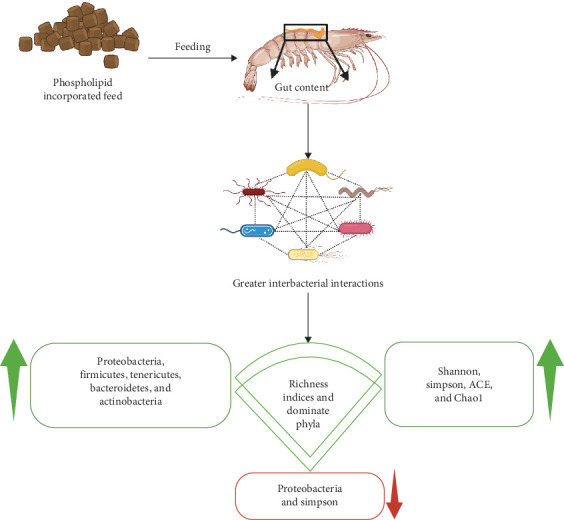
Mode of action of dietary phospholipids in the gut microbiota of crustaceans. The figure illustrates the impacts of phospholipid-enriched feed on the gut microbiota of crustaceans. It exhibits that PL increases the diversity and richness of gut bacteria, which leads to greater interbacterial interactions and a shift in the dominant phyla towards Proteobacteria. ACE, abundance-based coverage estimator, PL, phospholipid. The figure was generated using Biorender.com (Agreement number: TR277M0QQD).

The mode of action of PL in the crustacean gut microbiome, as illustrated in Figure 8, has been investigated using alpha diversity indices, with studies reporting positive influence of PL-enriched diets. For example, in *C. quadricarinatus* both ACE and Chao1 indices were highest with 3% SL inclusion, while the Shannon index peaked at a 2% SL level, though notably, the Simpson index decreased with increasing SL inclusion [26]. Similar positive effects of PL enrichment on alpha diversity indices were also observed using higher dietary PL in *L. vannamei* (4% KO and 6%) and *P. clarkii* (2% SL) [5, 95].

In a study on *L. vannamei*, 2005 OTUs were identified, among which 163 OTUs were common between the control and PL-fed groups, representing 8.13% of the total. Among the major types of PL-enriched feed (KO, EL, and SL), KO supplementation led to a higher abundance of Fusibacter, a bacterium linked to antioxidant activity and immune function. This indicates that food PL can induce microbial diversity through the regulation of iron metabolism, which could boost immune resilience in female *L. vannamei* [5]. In another study on *P. clarkii*, a total of 30 phyla, 60 classes, 118 orders, 232 families, and 476 genera were isolated. Specifically, the relative abundance of Tenericutes was significantly different at varying levels of PL, while the lowest Firmicutes/Bacteroidetes ratio was observed with 6% PL supplementation. Furthermore, increasing dietary PL levels reduced the abundance of *Shewanella* but significantly increased that of *Citrobacter* [44].

The phylum Proteobacteria can be both pathogenic (reducing the growth performance of crustaceans) and beneficial, playing roles in biogeochemical processes and carbon, nitrogen, and sulfur cycling [162, 163]. Although it occurs as a benign microbe to the gut microbiome, any malfunctioning in the microbiome community can lead to its rapid proliferation, endangering shrimp production [154, 164]. As such, the effect of PL-levels on Proteobacteria abundance, exhibiting the species-specific nature, can have variable impacts. For instance, while higher PL levels have been shown to reduce Proteobacteria abundance in *C. quadricarinatus* and *L. vannamei*, they have been observed to increase it in *P. clarkii* [5, 26, 44].

Firmicutes and Bacteroidetes play a crucial role in crustacean health, contributing to gut immune homeostasis by promoting both immune responses and fermentation processes, which generate energy for physiological functions [5, 162, 165, 166]. Similarly, the Actinobacteria phylum is considered beneficial due to its role in enhancing crustacean immune function. The phylum is linked to various immune parameters like T-AOC, total nitric oxide synthase, HSP70, Trx, Lys, proPO, Muc-1, Muc-2, and Muc-5AC [166]. Consistent with these benefits, studies have shown the positive effects of PL-enriched diets on these bacteria phyla in *C. quadricarinatus*, *L. vannamei*, and *P. clarkii* [5, 26, 44].

The mechanism of PL influence extends to direct nutrient provision for gut microbes, potentially altering their composition and activity. For example, increased PL can enhance the activity of microbiome genes involved in lipid and energy metabolism, leading to greater interbacterial interactions. These interactions are reflected in observed changes like a reduced abundance of Proteobacteria alongside increases in Firmicutes, Actinobacteria, Tenericutes, and Bacteroidetes. Such shifts in bacterial populations suggest complex ecological niche processes and bacterial symbiosis influenced by dietary PL [167].

## 11. PL and Osmoregulation

### 11.1. Effects of PL on Osmoregulation in Crustacean

Osmoregulation is the process by which organisms maintain a balance of dissolved substances (solutes) within and outside their cells (in the interstitial fluid) for proper function [168]. Aquatic crustaceans, through osmoregulation, can tolerate a wide range of salinities in their environment. This is achieved by controlling the osmotic pressure of their hemolymph through the regulation of hemolymph osmolytes [169]. The enzyme Na+/K+-ATPase (NKA) plays a crucial role in maintaining this osmotic pressure [170]. NKA is a member of the P-type ATPase family and is located in the cell membrane of various tissues and cells [171]. The NKA pump aids in preserving membrane potential and osmotic balance within cells [170].

NKA activity facilitates the movement of potassium (K^+^) and sodium (Na^+^) ions across the cell membrane against their concentration gradients. It maintains a higher concentration of potassium intracellularly and a higher concentration of sodium extracellularly. Several studies suggest that suitable dietary PL levels could play a significant role in maintaining NKA activity and, consequently, the osmoregulation process in aquatic organisms [172–174]. However, research on specific PL requirements for osmoregulation in crustacean aquaculture is limited. This highlights a gap in our knowledge regarding the impact of PL on NKA activity and osmoregulation.

Furthermore, existing research on the effect of PL on NKA activity presents contradictory findings. For example, a study on *S. paramamosain* observed a decreasing trend in the NKA mRNA expression with increasing dietary PL levels, with the lowest expression at 4% PL and the highest in the PL-free diet [19]. Conversely, another study on *S. paramamosain* reported a significant increase in NKA activity with increasing PL inclusion, with 4% EL yielding the highest activity and 0.1% the lowest [57]. These findings emphasize the need for further research to understand the complex relationship between PL and NKA activity in crustaceans. Changes in environmental salinity can trigger modifications in NKA activity, allowing crustaceans to adapt to varying osmotic conditions and survive in diverse aquatic environments [175]. Therefore, future studies must rigorously control environmental salinity as a potential confounding factor when evaluating the impact of PL impact on NKA activity and osmoregulation.

## 12. Conclusions and Future Outlook

Ensuring the health and development of cultured crustaceans is critical for a sustainable aquaculture industry, and nutrition plays a vital role. PL have emerged as a powerful nutritional supplement due to their support in various physiological processes, including growth, metabolism, reproduction, immunity, gut microbiota, and osmoregulation. Dietary PL supplementation offers a promising strategy to enhance the overall performance and well-being of crustaceans in aquaculture. Studies have shown that PL can stimulate the production of important essential FAs like DHA and n-3 PUFA, further improving antioxidant capacity and general physiological function.

While this review highlights the significance of incorporating PLs into crustacean diets, knowledge gaps persist. Further research utilizing purified diets and various PL sources is imperative to fully elucidate the physiological basis for PL requirements and how PL classes and FA composition influence their efficacy. This study lacks a species-specific comparison in terms of the different parameters used in this study. Future research should be focused on analyzing a more systematic comparison of differences in PL responses among species. Again, future studies should also incorporate detailed cellular and molecular studies to delineate the mechanistic pathways through which PL influence crustacean physiology. Understanding gene expression and cellular signaling involving PL metabolism will provide insights into their biological aspects of growth, immunity, and osmoregulation. Furthermore, in vivo, studies on the role of PL supplementation on cellular stress responses would improve the understanding of their potential therapeutic use in aquaculture.

Additionally, research should determine the ideal dietary PL supply for varied species and developmental stages, while also examining how PL requirements interact with other dietary components, such as protein and EFAs, to optimize overall nutrition. Again, this study examined generic trends across different PL dosages even though the administration quantities are extensive. Analysis of meta-regression data alongside creation of an S-shaped response curve will help identify specific turning points which should be studied in the future. By addressing these knowledge gaps, researchers and aquaculturists can develop effective PL supplementation strategies tailored to specific crustacean needs, contributing to a more sustainable and productive aquaculture industry with improved animal health, performance, and disease resistance.

## Figures and Tables

**Table 1:** Keywords and search phrases selection list.

Phospholipids	Crustacean	Indicators
Egg lecithin, soy lecithin, krill oil lecithin	Crab, prawn, shrimp, lobster, Chinese mitten crab, *Penaeus monodon*, *Cherax quadricarinatus*, *Procambarus clarkii*, *Scylla paramamosain*, *Portunus trituberculatus*, *Litopenaeus vannamei*, *Eriocheir sinensis*	Growth, diet, metabolism, reproduction, impact, immune parameters, higher crustacean production yield, dietary phospholipids, NKA activity, gut microbes, GSI reproduction indicator, growth mechanisms, factors affecting growth, development, immunity of crustaceans, phospholipids supplementation, aquaculture, growth of crustaceans fed with phospholipid, malondialdehyde (MDA) in crustaceans, immunity in crustaceans, humoral immunity of crustacean, osmoregulation of phospholipid-fed crustaceans, gut microbes of crustacean fed with phospholipid, PL supplementation on crustacean HDL, dietary PL in crustacean lipid metabolism, dietary PL role in crustacean osmoregulation etc.

**Table 2:** Phospholipid composition (% of wet sample) in major sources of phospholipids.

Component	Percentage (% of wet sample)	References
Soy lecithin		
Total Phospholipid (PL)	38–45	[29]
Phosphatidylcholine (PC)	16.83–22.23	[30]
Phosphatidylethanolamine (PE)	10.0–13.67	[30]
Phosphatidylinositol (PI)	14.66–17.27	[30]
Phosphatidic acid (PA)	5.28–8.57	[30]
Lysophosphatidylcholine (LPC)	0.58	[31]
Egg yolk lecithin		
Phosphatidylcholine (PC)	66–67	[32]
Phosphatidylethanolamine (PE)	15–24	[32]
Phosphatidic acid (PA)	0.9	[33]
Phosphatidylinositol (PI)	0.6	[33]
Phosphatidylserine (PS)	1	[32]
Lysophosphatidic acid (LPA), lysophosphatidylethanolamine (LPE) and sphingomyelins (SM)	3–6	[32, 33]
Sphingomyelin (SM)	2.5	[33]
Krill oil		
Phospholipid (PL)	24.7	[34]
Phosphatidylcholine (PC)	87.93–95.16	[35]
Phosphatidylethanolamine (PE)	7.00	[36]
Phosphatidylserine (PS)	5.10	[36]
Lysophosphatidylcholine (LPC)	43.31	[37]

**Table 3:** Comparative assessment of nutritional roles, economic feasibility, and sustainability of major phospholipid sources in crustacean diets.

PL source	Advantages	Disadvantages	Economic viability	Supply stability	Sustainability	Reference
Soy lecithin	Inexpensive, widely available, good emulsifier, and rich in phospholipids	Possible genetically modified organisms (GMO) concerns and lower purity than animal-derived lecithin	Highly cost-effective and ideal for mass production	Very stable and consistent due to large-scale soybean farming	Generally sustainable, but concerns over deforestation and GMO practices exist	[64, 65]

Egg lecithin	High-purity phospholipids with excellent emulsifying and health-promoting properties	High extraction cost and lower yield compared to plant-based lecithin	Less viable for large-scale production due to costly raw materials and processing	Dependent on egg industry byproducts, with limited and variable availability	Less sustainable than plant sources, but reusing egg-processing waste adds some value	[33, 38]

Krill oil lecithin	High bioavailability of phospholipids and rich in omega-3 (EPA & DHA)	Expensive and limited supply due to reliance on Antarctic krill	Less viable for bulk use	Seasonal and limited harvest, influenced by environmental and regulatory factors	Concerns over krill harvesting impacting marine ecosystems	[28, 66]

**Table 4:** Fatty acid profile of soy lecithin, egg yolk lecithin, and krill oil.

Fatty Acid	Soy lecithin [31]	Egg yolk lecithin [33]	Krill oil [28]
Myristic acid (C14:0)	—	0.23	5.0–13.0
Pentadecanoic acid (C15:0)	—	0.10	—
Heptadecanoic acid (C17:0)	—	0.33	—
Palmitic acid (C16:0)	23.4	19.44	17.0–24.6
Palmitoleic acid (C16:1)	—	1.09	2.5–9.0
Stearic acid (C18:0)	20.4	7.72	—
Cis-11-octadecenoic acid (C18:1)	—	—	4.7–8.1
Oleic acid (C18:1 n-9)	18.4	57.80	—
Elaidic acid (C18:1 n-9)	—	—	6.0–14.5
Linoleic acid (C18:2 n-6)	53.3	7.45	ND–3.0
Linolenic acid (C18:3 n-3)	4.81	1.67	0.1–4.7
Arachidonic acid (C20:4 n-3)	1.37	0.83	—
EPA (EPA) (C20:5 n-3)	—	—	14.3–28.0
Docosapentaenoic Acid (DPA) (C22:5 n-3)	—	—	ND–0.07
Docosahexaenoic acid (DHA) (C22:6 n-3)	—	2.62	7.1–15.7

**Table 5:** A summary of recent research on the impact of PL diet on crustacean growth.

Species	Inclusion rate (percent)	Duration (days)	Parameter	Results		Study
				Positive	Negative/neutral	
*Penaeus monodon*	1, 1.5, 2, and 2.5	60	FBW, RGR, FCR, PER, and SR	– **FBW:** Highest- from 3.28 g to 14.12 g (2.5% SL) – **RGR:** Highest- 6.20% (2.5% SL); lowest- 5.04% (1% SL) – **PER:** Highest- 1.57 (1.5% SL); lowest- 1.38 (1% SL) – **SR:** Highest- 82.22% (2% and 2.5% SL); lowest- 75.56 (1% and 1.5%)	– **FCR:** Lowest- 2.11 (2.5% SL); highest- 2.33 (1% SL)	[18]

*Scylla paramamosain*	0^a^, 1, 2, and 4	56	SR, FBW, WG, MF, and SGR	– **WG:** Highest- 3267.41% (2% SL); lowest- 2749.45% (0% SL), – **FBW:** Highest- 1.35 g (2%SL); lowest- 1.14 (0% SL) – **SGR:** Highest- 6.26% (2% SL); lowest- 5.97 (0% SL) – **MF:** Highest- 4.97 (4% SL); lowest- 4.68 (1% SL)	– **SR:** Lowest- 86.90 (2% SL); highest- 92.86 (0% SL)	[19]

*Portunus trituberculatus*	0, 1, 2, and 4	56	FBW, WG, SGR, FCR, SR, PER, and MF	– **FBW:** From 3.69 g to 44.48 g (4% SL) – **WG:** Highest- 1105.58% (4% SL); lowest- 937.62% (0% SL) – **SGR:** Highest- 4.70 (4% SL); lowest- 4.41% (0% SL) – **MF:** Highest- 3.29 (4% SL); lowest- 2.76 (0% SL) – **PER:** Highest- 1.75 (4% SL); lowest- 1.26 (0% SL) – **SR:** Highest- 88.33% (4% SL); lowest- 71.67% (0% SL)	– **FCR:** Lowest- 1.28 (4% SL); highest- 1.78 (0% SL)	[43]

*Procambarus clarkii*	0, 2, and 6	60	WG, FBW, SGR SR, and MF	– **FBW:** From 5.90 g to 10.48 g (6% SL) – **WGR:** Highest- 79.76% (6% SL); lowest- 53.35% (2% SL) – **SR:** Highest- 100% (6% SL); lowest- 93.33% (0% SL)	– **MF:** Optimum molting (6% SL)	[44]

*Litopenaeus vannamei* ^b^	4	28	SR, WG, CF, and SGR	– **WG:** Highest- ~40% (4% KO); lowest- ~25% (0%) – **SGR:** Highest- ~1.20% (4% KO); lowest- ~0.8% (0%)	– **SR and CF:** No significant effects	[5]

*Litopenaeus vannamei*	0, 2, and 4	56	SR, FBW, SGR, and FCR	– **SR:** Highest- 94% (4% SL); lowest- 86% (0% SL) – **FBW:** Highest- 10.61 g (4% SL); lowest- 10.32 g (2% SL) – **SGR:** Highest- 6.71% (4% SL); lowest- 6.58% (0% SL)	– **FCR:** Lowest- 1.35 (2% SL); highest- 1.40 (0% and 4% SL)	[11]

*Portunus trituberculatus*	0.99,1.65, 2.51, 3.63, 4.95, and 6.12	128	FBW, WG, SGR, FE,SR, and SI	– **FBW:** Highest- 92.91 g (3.63%); lowest- 72.71 g (0.99%) – **WG:** Highest- 266.33% (3.63%); lowest- 185.35% (0.99%) – **SGR:** Highest- 2.32% (3.63%); lowest- 1.87% (0.99%) – **WG:** Highest- 266.33% (3.63%); lowest- 185.35% (0.99%) – **HSI:** Highest- 6.62% (4.95%); lowest- 5.54% (0.99%) – **SR:** Highest- 80% (3.63%); lowest- 75.55% (0.99% and 6.92%)	– **FE:** No significant impact. Value ranged between 0.5 to 0.54	[13]

*Eriocheir sinensis* ^b^	0, 1 and 3	56	FBW, SR, WG, SGR, HSI, and MF	– **FBW:** Highest- 1.95 g (3% KO); lowest- 1.02% (0%) – **SR:** Highest- 95.24% (3% SL); lowest- 80.95% (0%) – **WG:** Highest- 647.83% (3% KO); lowest- 288.73% (0%) – **SGR:** Highest- 3.59% (3% KO); lowest- 2.42% (0%) – **MF:** Highest- 2.78 (3% KO); lowest- 1.91 (0%)	– **HSI:** Lowest- 8.19% (3% KO); highest- 8.56% (0%)	[45]

*Scylla paramamosain* ^c^	0, 1, 2, 3, and 4	56	SR, FBW, WG, SGR, and MF	– **SR:** Highest- 92.86% (3%); lowest- 42.86% (0%) – **FBW:** Highest- 0.54 g (2%); lowest- 0.37 g (0%) – **WG:** Highest- 6590.46% (2%); lowest- 4393.13% (0%) – **SGR:** Highest- 7.49% (2%); lowest- 6.78% (0%)	– **MF:** Lowest- 5.43% (4%); highest- 6.05% (2%)	[46]

*Portunus trituberculatus*	0, 1, and 2	56	FBW, SGR, WG, MF, FCR, PER, and HIS	– **FBW:** Highest- 19.52 g (2%); lowest- 14.34 g (0%) – **SGR:** Highest- 3.42% (2%); lowest 2.83% (0%) – **WG:** Highest- 456.63% (2%); lowest- 311.02% (0%) – **MF:** Highest- 1.60% (2%); lowest 1.46% (0%) – **FCR:** Highest- 1.07% (0% along with 0.2% CHO and 2%); lowest- 0.93% (0%) – **PER:** Highest- 2.01% (2%); lowest- 1.76% (0%) – **HSI:** Highest- 4.03% (2%); lowest- 2.51% (0%) highest- 3.59% (3% KO); lowest- 2.42% (0%) highest- 3267.41%	—	[47]

^a^0% refers to the control of the feeding trials. PL were sourced from SL except, ^b^where EL and KO were used as additional sources, and ^c^where EL was used as the only source.

**Table 6:** A summary of recent research on the impact of PL diet on lipid metabolic parameters of crustaceans.

Species	Inclusion rate (percent)	Duration (days)	Metabolic parameters	Result		Study
				Positive	Negative/Neutral	
*Cherax quadricarinatus* ^a^	2	70	TG, T-CHO, HDL, and LDL	– **TG:** Highest- ~0.21 mmol/gprot (2% KO); lowest- ~0.17 mmol/gprot (0%) – **T-CHO:** Highest- ~1.25 mmol/gprot (2% KO); lowest- ~0.75 mmol/gprot (0%) – **HDL:** Highest- ~0.14 mmo(l) (L) (2% EL); lowest- ~0.075 mmo(l) (L) (0%)	– **LDL:** Lowest- ~0.25 mmol L (2% KO); highest- ~0.375 mmol L (0%)	[20]

*Scylla paramamosain* ^b^	0.1, 0.5, 1, 2, 4 (EL), and 2 (SL)	60	TG and T-CHO	– **TG:** Highest- 5.89 mmol/gprot (4% EL); lowest- 2.80 mmol/gprot (0.5% EL) – **T-CHO:** Highest- 1.25 mmol/gprot (4% EL); lowest- 0.49 mmol/gprot (0.1% EL)		[57]

*Litopenaeus vannamei* ^a^	0^c^ and 4	28	TG, LDL, T-CHO, and VLDL	– **TG:** Highest- ~0.55 mmol/L (4% KO); lowest- ~0.4 mmol/L (0%) – **LDL:** Highest- ~1.1 mmol/L (4% KO); lowest- ~0.85 mmol/L (0%) – **T-CHO:** Highest- ~1.70 mmol/L (4% KO); lowest- ~0.65 mmol/L (0%) – **VLDL:** Highest- ~1 μmol/mL (4% KO); lowest- ~0.6 μmol/mL (0%)		[24]

*Scylla paramamosain*	0.5, 1.0, and 1.5	56	T-CHO, LDL, HDL, and TG	– **T-CHO:** Highest- 1.43 mmol/L (1.5%); lowest-0.71 mmol/L (0.5%) – **TG:** Highest- 0.135 mmol/L (1.5%); lowest- 0.065 mmol/L (0.5%)	– **LDL:** Lowest- 0.02 mmol L (1.5%); highest- 0.076 mmol/L (0.5%) – **HDL:** Lowest- 0.01 mmol/L (1.5%); highest- 0.03 mmol/L (1%)	[22]

*Cherax quadricarinatus*	0, 1, 2, and 3	56	T-CHO, TG, and Lipid metabolism enzyme (ACC and CPT-1)	– **T-CHO:** Highest- ~2.8 mmol/gprot (1%); lowest- ~1 mmol/gprot (0%) – **TG:** Highest- ~2.9 mmol/gprot (2%); lowest- ~0.75 mmol/gprot (0%) – **Lipid metabolism enzyme** – **CPT-1:** Highest- ~9.5 ng/mL (3%); lowest- ~8.1 ng/mL (0%)	– **Lipid metabolism enzyme** – **ACC:** Lowest- ~1.8 ng/mL (2%); highest- ~2.1 ng/mL (0%)	[26]

*Eriocheir sinensis* ^a^	0 and 2.5	70	TG, T-CHO, LDL, HDL, and VLDL	– **TG:** Highest- ~0.21 mmol/L (2.5% KO); lowest- ~0.9 mmol/L (0%) – **T-CHO:** Highest- ~1.6 mmol/L (2.5% KO); lowest- ~0.75 mmol/L (0%) – **LDL:** Highest- ~2.1 mmol/L (2.5% KO); lowest- ~2 mmol/L (2.5% EL) – **HDL:** Highest- ~4.2 mmol/L (2.5% SL); lowest- ~3.9 mmol/L (0%) – **VLDL:** Highest- ~2 mmol/L (2.5% KO); lowest- ~1.5 mmol/L (0%)		[23]

*Procambarus clarkii*	0, 2, and 6	60	TG, T-CHO, LDL, and HDL	– **TG:** Highest- 0.24 mmol/L (6%); lowest- 0.11 mmol/L (2%) – **T-CHO:** – **Serum:** Highest- 0.56 mmol/L (6%); lowest- 0.21 mmol/L (0%) – **Hepatopancreas:** Highest- 0.62 mmol/L (6%); lowest- 0.34 mmol/L (0%) – **LDL:** Highest- 0.045 mmol/L (6%); lowest- 0.023 mmol/L) (0%) – **HDL:** Highest- 0.18 mmol/L (0%); lowest- 0.12 mmol/L (0%)		[95]

*Eriocheir sinensis*	1 and 5	56	VLDL	– **VLDL** – **Hepatopancreas:** Highest- 4.11 (5%); lowest- 2.04 (1%) – **Serum:** Highest- 2.19 (5%); lowest- 1.41 (1%)		[94]

*Portunus trituberculatus*	0.99, 1.65, 2.51, 3.63, 4.95, and 6.12	112	TP, T-CHO, TG, HDL, LDL, and Lipid metabolism enzyme (PLTP, PCT, PLA2 and FAS)	– **T-CHO:** Highest- 4.26 mmol/L (2.51%); lowest- 3.15 mmol/L (0.99%) – **LDL:** Highest- 2.36 mmol/L (3.63%); lowest- 2.07 mmol/L (0.99%) – **Lipid Metabolism Enzyme** – **PCT:** 105.18 U/mgprot (3.63%); lowest- 38.03 U/mgprot (1.65%) – **PLA2:** 47.37 U/mgprot (3.63%); lowest- 9.40 U/mgprot (0.99%)	– **TG:** Lowest- 2.12 mol/L (6.12%); highest- 2.73 mmol/L (2.51%) – **HDL:** Lowest- 1.81 mmol/L (4.95%); highest- 1.92 mmol/L (2.51%) – **Lipid Metabolism Enzyme** – **PLTP:** Lowest- 3.80 μg/mgprot (6.12%); highest- 16.83 μg/mgprot (3.63%) – **FAS:** Lowest- 3.41 U/mgprot (6.12%); highest-4.85 U/mgprot (3.63%)	[13]

*Portunus trituberculatus*	0, 1, and 2	56	HDL, TG, and T-CHO	– **Serum** – **HDL:** Highest 0.22 mg/dl (2%); lowest- 0.11 mg/dl) (0%) – **T-CHO**: Highest 5.84 mg/dl (2%); lowest 3.57 mg/dl) (0%) – **Whole body** – **T-CHO:** Highest- 2.59 μg/mg (1%); lowest- 2.16 μg/mg (**0% and 1%**) – **Hepatopancreas** – **T-CHO:** Highest- 13.20 μg/mg (2%); lowest- 7.96 μg/mg (0%) – **Muscle** – **T-CHO:** Highest- 3.91 μg/mg (1%); lowest- 2.72 μg/mg (0%)	– **Serum** – **TG:** Lowest- 1.84 mg/dl (2%); highest- 3.10 mg/dl (1%)	[47]

*Note:* All phospholipids were sourced from soy lecithin, except where additional sources include ^a^krill oil and egg yolk lecithin or ^b^egg yolk lecithin; ^c^0% refers to control of the feeding trials.

**Table 7:** A summary of recent research on the impact of PL diet on crustacean nutrition.

Species	Inclusion Rate (percent)	Duration (day)	Parameters	Results		Study
				Positive	Neutral/Negative	
*Scylla paramamosain* ^a^	0.1, 0.5, 1, 2, 4 (EL), and 2 (SL)	60	SFA, MUFA, PUFA, and Lc-PUFA	– **SFA:** Highest- 46 (EL 4%); lowest- 30 (EL 0.1%) (Approximately) – **MUFA:** Highest- 22 (EL 1%); lowest- 18 (EL 0.5%) (Approximately) – **Lc-PUFA:** Highest- 18 (EL 4%); lowest- 12 (EL 0.1%) (Approximately)	– **PUFA:** Lowest- 10 (EL 4%); highest- 29 (EL 0.1%) (Approximately)	[57]

*Procambarus clarkii*	0, 2, and 6	60	SFA, MUFA, PUFA, HUFA, n-3 PUFA, n-6 PUFA, DHA, and EPA	– **SFA:** Highest- 24.03 (6%); lowest- 20.76 (0%) – **MUFA:** Highest- 39.22 (6%); lowest- 34.15 (0%) – **DHA:** Highest- 0.53 (2 + CH 10%); lowest- 0.37 (2% + CH 5%) – **EPA:** Highest- 1.78 (6%); lowest- 0.86 (2%)	– **PUFA:** Lowest- 31.66 (6%); highest- 41.68 (0%) – **n-3 PUFA:** Lowest- 3.82 (6%); highest- 5.12 (0%) – **n-6 PUFA:** Lowest-27.84 (6%); highest- 36.65 (0%)	[95]

*Scylla paramamosain* ^a^	0, 1, 2, 3, and 4	56	SFA, MUFA, PUFA, Lc-PUFA, DHA, and EPA	– **PUFA:** Highest- 21.04 (4%); lowest- 11.25 (2%) – **DHA:** Highest- 11.18 (2%); lowest- 10.37 (0%)	– **SFA:** Lowest- 31.71 (4%), highest- 35.53 (2%) – **MUFA:** Lowest- 19.74 (3%); highest- 22.93 (0%) – **Lc-PUFA:** Lowest- 20.55 (3%); highest- 22.05 (2%) – **EPA:** Lowest- 8.02 (3%); highest- 8.49 (2%)	[53]

*Eriocheir sinensis* ^a,b^	0, 1, and 3	56	SFA, MUFA, PUFA, HUFA, n-3 PUFA, n-6 PUFA, DHA, and EPA	– **SFA:** Highest- 32.36 (EL 3%); lowest- 28.99 (SL 3%) – **PUFA:** Highest- 19.98 (SL 3%); lowest- 10.80 (KO 3%) – **HUFA:** Highest- 11.63 (KO 3%); lowest- 4.85 (0%) – **n-3 PUFA:** Highest- 12.14 (KO 3%); lowest- 5.03 (0%) – **n-6 PUFA:** Highest- 19.67 (SL 3%); lowest- 10.30 (KO 3%) – **EPA:** Highest- 4.59 (KO 3%); lowest- 1.53 (0%) – **DHA:** Highest- 5.17% (KO 3%); lowest- 2.63 (0%)	– **MUFA:** Lowest- 45.22 (SL 3%); highest- 53.85 (0%)	[45]

*Scylla paramamosain*	0, 1, 2, and 4	56	SFA, MUFA, PUFA, Lc-PUFA, DHA, and EPA	– **SFA:** Highest- 27.59 (4%); lowest- 24.23 (0%) – **Lc-PUFA:** Highest- 21.98 (4%); lowest- 18.62 (0%) – **DHA:** Highest- 10.68 (4%); lowest- 9.80 (0%) – **EPA:** Highest- 8.98 (4%); lowest- 7.57 (0%)	– **MUFA:** Lowest- 29.24 (1%); highest- 32.02 (0% – **PUFA:** Lowest- 19.27 (4%); highest- 24.02 (0%)	[19]

*Portunus trituberculatus*	0, 1, and 2	56	SFA, MUFA, n-3 PUFA, n-6 PUFA, Lc-PUFA, DHA, and EPA	– **MUFA:** Highest- 219.4 g (/kg (2%); lowest- 185.4 g/kg (0%) – **n-6 PUFA:** Highest- 134.8 g/kg (1%); lowest- 112.2 g/kg (0%) – **DHA:** Highest- 136.5 g/kg (1%); lowest- 122.7 g/kg (0%)	– **SFA:** Lowest- 275.2 g /kg (2%); highest- 312.5 g /kg (0%) – **n-3 PUFA:** Lowest- 269.0 g/kg (2%); highest- 290.9 g /kg (1%) – **Lc-PUFA:** Lowest- 264.6 g/kg (2%); highest- 285.6 g /kg (1%) – **EPA:** Lowest- 130.3 g /kg (SL 0% + CH 0.8%); highest- 144.6 g/kg (0%)	[47]

*Portunus trituberculatus*	0, 1, 2, and 4	56	SFA, MUFA, PUFA, HUFA, n-3 PUFA, n-6 PUFA, DHA, and EPA	– **SFA:** Highest- 25.70 (4%); lowest- 23.45 (0%) – **HUFA:** Highest- 11.08 (4%); lowest- 8.40 (1%) – **n-3 PUFA:** Highest- 11.07(4%); lowest- 9.88(1%) – **EPA-** Highest- 3.74 (4%); lowest- 2.33 (1%) – **DHA-** Highest- 6.39 (4%); lowest- 5.65 (2%)	– **MUFA:** Lowest- 31.67 (2%); highest- 34.62 (0%) – **PUFA:** Lowest- 29.78 (4%); highest- 35.05 (2%) – **n-6 PUFA:** Lowest- 29.78 (4%); highest- 33.30 (1%)	[43]

*Note:* All diets contain soy lecithin. Superscript alphabet letter indicates additional phospholipids sources ^a^egg yolk lecithin; ^b^krill oil lecithin. 0% refers to control of the feeding trials.

**Table 8:** A summary of recent research on the impact of PL diet on the immune parameters of crustaceans.

Species	Inclusion rate (percent)	Duration (day)	Parameter	Result		Study
				Positive	Negative/Neutral	
*Cherax quadricarinatus* ^a,b^	2 (each)	70	SOD, T-AOC, GSH-Px, and MDA	– **SOD:** Highest- ~17 U/mL (2% KO); lowest- ~12 U/mL (Control) – **T-AOC:** Highest- ~0.5 mM (2% KO); lowest- ~0.3 mM (Control) – **GSH-Px:** Highest- ~220 mol/mL (2% KO); lowest- ~160 mol/mL (Control)	– **MDA:** Lowest- ~5 nmol/mgprot (2% KO); highest- ~13 nmol/mgprot (Control)	[20]

*Penaeus vannamei* ^b^ *⁣* ^*∗*^	0, 2, 4, and 6	56	proPO, SOD, proPO activating enzyme, serine protease, beta-glucan binding protein, and hemocyanin gene	– **proPO:** Upregulation (4%) – **SOD:** Upregulation (6%) – **proPO activating enzyme:** Upregulation (6%) – **Serine protease:** Upregulation (6%) – **Beta-glucan binding protein:** Upregulation (2%) – **Hemocyanin gene:** Upregulation (4%)		[21]

*Scylla paramamosain*	0.5, 1.0, and 1.5	14	T-AOC, MDA, GST, GSH-Px, and SOD	– **T-AOC:** Highest- 2.37 Umg/prot (1%); lowest- 0.85 Umg/prot (0.5%) – **MDA:** Highest- 5.76 nmolmg/prot (1%); lowest- 1.95 nmolmg/prot (0.5%) – **GST:** Highest- 52.21 U/mgprot (1.5%); lowest- 22.58 U/mgprot (0.5%) – **GSH-Px:** Highest- 115.58 U/mgprot (1.5%); lowest- 34.85 U/mgprot (1.5% SL + 1.5% Cholesterol) – **SOD:** Highest- 75.97 U/mgprot (1.5%); lowest- 31.32 U/mgprot (1%)		[22]

*Procambarus clarkii*	0, 2, and 6	60	SOD, MDA, CAT, GSH-Px, T-AOC, LZM, AKP, and ACP	– **SOD:** Highest- 819.87 U/mL (6%); lowest- 735.08 U/mL (0%) – **MDA:** Highest- 2.01 nmol/mL (6%); lowest-0.64 nmol/mL (0%) – **GSH:** Highest- 132.98 U/mL (2%); lowest- 47.06 U/mL (6% + 1% Cholesterol) – **LZM:** Highest- 123.53 U/mL (2%); lowest- 78.43 U/mL (0%)	– **CAT:** Lowest- 1.18 U/mL (6%); highest- 2.08 U/mL (0%) – **T-AOC:** Lowest- 0.28 mmol/L (6%); highest- 0.4 mmol/L (0%) – **AKP:** Lowest- 12.26 U/L (6%); highest- 35.42 U/L (0%) – **ACP:** Lowest- 15.92 U/L (6%); highest- 96.47 U/L (0%)	[44]

*Litopenaeus vannamei* ^a,b^	0 and 4	28	T-AOC, MDA, GSH-Px and SOD, PO, and LZM	– **T-AOC:** Highest- ~1.5 mmol /g (4% KO); lowest- ~0.75 mmol /g (0%) – **GSH-Px:** Highest- ~8.5 μmol/mgprot (4% KO); lowest- ~4 μmol/mgprot (0%) – **PO:** Highest- ~35 U (L) (4% KO); lowest- ~10 U (L) (0%) – **LZM:** Highest- ~5 U/L (4% KO); lowest- ~1 U/L (0%) – **SOD:** Highest- ~120 U/mgprot (4% KO); lowest- ~75 U/mgprot (0%)	– **MDA:** Lowest- ~50 nmol/mgprot (4% KO and EL); highest- ~140 nmol/mgprot (0%)	[5]

*Scylla paramamosain* ^a^	0, 1, 2, 3, and 4	56	SOD and MDA	– **SOD:** Highest- 80 U/mgprot (3%); lowest- 25 U/mgprot (0%)	– **MDA:** Lowest- 1 nmol/mgprot (4%); highest- ~2.75 nmol/mgprot (0%)	[53]

*Eriocheir sinensis* ^a,b^	0 and 2.5	70	GSH-Px and CAT, MDA, and SOD	– **GSH-Px:** Highest- ~60 μmol/mgprot (2.5% KO); lowest- 20 μmol/mgprot (0%) – **CAT:** Highest- ~7 U/mgprot (2.5% KO); lowest- ~0 U/mgprot (0%) – **SOD:** Highest- ~35 U/mgprot (2.5% KO); lowest- ~20 U/mgprot (0%)	– **MDA:** Lowest- ~2 nmol/mgprot (0%); highest- ~3.5 U/mgprot (2.5% KO)	[23]

*Scylla paramamosain*	0, 1, 2, and 4	56	MDA and SOD	– **SOD:** Highest- 150.84 U/mgprot (2%); lowest- 80.52 U/mgprot (0%)	– **MDA:** Lowest- 3.11 U/mgprot (1%); highest- 9.40 U/mgprot (0%)	[17]

*Litopenaeus vannamei*	0, 2, and 4 PL	56 days; 48 h (toll-like receptor mRNA; IMD mRNA, LZM mRNA)	SOD, LZM, toll-like receptor mRNA, IMD mRNA, LZM mRNA	– **SOD:** Highest- 418.96 unit/mL (4%); lowest- 317.81 unit/mL (0%) – **Toll-like receptor mRNA:** Highest- 1.38 (4%); lowest- 0.75 (0%) – **LZM mRNA:** Highest- 2.56 (4%); lowest- 0.42 (0%)	– **LZM:** Lowest- 111.71 unit/mL (0%); highest- 152.06 unit/mL (0% + 0.4% Cholesterol) – **IMD mRNA:** Lowest- 0.70 (0%); highest- 1.62 (0% + 0.2% Cholesterol)	[11]

*Eriocheir sinensis* ^a,b^	0, 1, and 3	56	GSH-Px, SOD and CAT, MDA	– **SOD:** Highest- 57.46 U/mgprot (3% KO); lowest- 32.90 U/mgprot (0%) – **CAT:** Highest- 10.70 U/mgprot (3% KO); lowest- 5.42 U/mgprot (0%) – **GSH-Px:** Highest- 64.10 U/mgprot (3% KO); lowest- 49.99 U/mgprot (0%)	– **MDA:** Lowest- 0.41 nmol/mgprot (3% KO); highest- 0.77 nmol/mgprot (0%)	[45]

*Portunus trituberculatus*	0.99, 1.65, 2.51, 3.63, 4.95, and 6.12	112	CAT, GSH, SOD, T-AOC, and MDA	– **GSH:** Highest- 251.34 μmol/gprot (3.63%); lowest- 136.07 μmol/gprot (0.99%) – **SOD:** Highest- 28.27 U/mgprot (3.63%); lowest- 19.54 U/mgprot (6.125) – **T-AOC:** Highest- 4.38 U/mgprot (4.95%); lowest- 2.07 U/mgprot (0.99%) – **MDA:** Highest- 7.02 nmol/mgprot (6.12%); lowest- 2.18 nmol/mgprot (0.99%)	– **CAT:** Lowest- 46.54 U/mgprot (6.12%); highest- 102.72 U/mgprot (3.63%)	[13]

*Portunus trituberculatus*	0, 1, 2, and 4	56	SOD, T-AOC, GSH-Px, MDA, and LZM	– **SOD:** Highest- 113.38 U/mgprot (4%); lowest- 72.13 U/mgprot (0%) – **GSH-Px:** Highest- 328.14 U/mgprot (4%); lowest- 225.95 U/mgprot (0%) – **T-AOC:** Highest- 8.20 U/mgprot (1%); lowest- 6.08 U/mgprot (0%) – **LZM:** Highest- 45.28 Ug/mgprot, (4%); lowest- 33.08 Ug/mgprot, (0%)	– **MDA:** Lowest- 9.12 nmol/mgprot (4%); highest- 13.71 nmol/mgprot (0%)	[43]

*Note:* All diets contain soy lecithin. Superscript alphabet indicates additional phospholipid sources ^a^egg yolk lecithin; ^b^krill oil lecithin, ^b^*⁣*^*∗*^indicates krill oil only. 0% refers to the control of the feeding trials.

**Table 9:** A summary of recent research on the impact of PL diet on the reproductive parameters of crustaceans.

Species	Inclusion rate (percent)	Duration (day)	Parameter	Result		Study
				Positive	Neutral/Negative	
*Cherax quadricarinatus* ^a,b^	0 and 2	70	GSI, ovarian histology, TG in ovary, and RHC	– **GSI:** Highest- ~3% (2% KO); lowest- ~2% (0%) – **Ovarian Histology:** Largest mature oocytes (2% KO) – **TG in Ovary:** Highest- ~0.5 mmol/gprot (2% KO); lowest- ~0.1% (0%) – **RHC:** ° **E2:** Highest- 60 ng/L (2% KO); lowest- 40 ng/L (0%) ° **MF:** Highest- ~125 ng/L (2% KO); lowest- ~85 ng/L (0%)	– **RHC:** ° **LH:** Lowest- ~1.5 ng/L (2% KO); highest- ~3.75 mIU /mL (0%) ° **GIH:** Lowest-~14 ng/L (2% KO); highest- ~22.5 ng /L (0%) ° **MIH:** Lowest- ~18 pg /mL (2% KO); highest- ~25 pg /mL (0%)	[20]

*Procambarus clarkii*	0, 2, and 6	60	GSI, RHC, and ovary morphological structure	– **GSI:** Highest- 5:83% (6%); lowest- 4.01% (0%) – **Ovary morphological structure:** More compactly arranged eggs and signified advanced ovarian developmental stages (6%)	– **RHC:** ° **E2:** Lowest- 14.50 ng/L (6%); highest- 19.08 ng /L (0%)	[95]

*Litopenaeus vannamei* ^a,b^	0 and 4	28	GSI, RHC, and ovarian development status	– **GSI:** Highest- 4.5% (4% KO); lowest- 2.5% (0%) – **RHC:** ° **E2:** Highest- ~65 ng/L (4% KO); lowest- ~38 ng /L (0%) ° **MF:** Highest- ~125 ng /L)(4% KO); lowest- ~80 ng /L (0%) – **Ovarian development status:** More effective yolk granule deposition (4% KO and 4% WL)	– **RHC:** ° **GIH:** Lowest- ~15 ng/L (2% KO); highest- ~19 ng /L (0%) ° **MIH:** Lowest- ~19 pg /mL (2% KO); highest- ~22 pg /mL (0%)	[24]

*Eriocheir sinensis* ^a,b^	0 and 2.5	70	GSI, ovary morphology, VGR mRNA and VG in ovaries, RHC, FA composition of ovary	– **GSI:** Highest- ~9% (2.5% KO); lowest- ~7% (0%) – **Ovary morphology:** Advanced ovarian development status was recorded with 4% krill oil – **VGR mRNA:** Highest- ~3 (2.5% KO); lowest- ~1 (0%) – **VG:** Highest- ~3 (2.5% KO); lowest- ~1 (0%) – **RHC:** – **PROG:** Highest- ~950 pg /mL (2.5% KO); lowest- ~750 pg /mL (0%) ° **E2:** Highest- ~60 pg/mL (2.5% KO); lowest- ~50 pg /mL (0%) – **FA composition of ovary:** Inclusion of 2.5% krill oil resulted in improved total Lc-PUFA and n-3 fatty acids		[23]

*Litopenaeus vannamei*	0, 2, 4, and 6	30	GSI, egg diameter, and VTG gene expression	– **Egg Diameter:** Highest- 106.7 μm (4% SL); lowest- 93.3 μm (6%) – **VTG mRNA Transcript:** Greatest relative abundance (6%)	– **GSI:** Lowest- 2.7% (6%); highest- 3.4% (4%)	[25]

*Portunus trituberculatus*	0.99, 1.65, 2.51, 3.63, 4.95, and 6.12	112	GSI, RHC, and VG gene expression	– **GSI:** Highest- 0.34% (3.63%); lowest- 0.28% (0.99%) – **VG Transcript:** Highest- ~11 (6.12%); lowest- ~1 (0.99) – **RHC:** – **PROG:** Highest- ~425 pg /mL (6.12%); lowest- ~275 pg /mL (0.99%) ° **E2:** Highest- ~45 pg /mL (6.12%); lowest- ~34 pg /mL (0.99%)	—	[13]

*Portunus trituberculatus*	0, 1, 2, 4, 6, and 8	112	VTG gene expression, FABP gene expression, RHC	– **VTG Gene Expression:** Highest- ~1.75 (4%); lowest- ~1 (0%) – **FABP Gene Expression:** Highest- ~7 (8%); lowest- ~1 (0%) – **RHC:** – **PROG:** Highest- ~420 pg /mL (8%); lowest- ~300 pg/mL (0%) ° **E2:** Highest- ~50 pg/mL (8%); lowest- ~36 pg /mL (0%)	—	[12]

*Note:* All diets were formulated using soy lecithin. Superscript alphabet indicates additional phospholipids sources ^a^egg yolk lecithin; ^b^krill oil lecithin. 0% refers to the control of the feeding trials.

**Table 10:** A summary of recent research on the impact of PL diet on crustacean gut microbes.

Species	Inclusion rate (percent)	Duration (days)	Parameter	Result		Study
				Positive	Negative/Neutral	
*Cherax quadricarinatus*	0, 1, 2, and 3	56	Richness index and dominant phyla	– **Richness index (Shannon):** Highest- 3 (2%); lowest- 1.9 (0%) – **Richness index (ACE):** Highest- 400 (3%); lowest- 250 (1%) – **Richness index (Chao1):** Highest- 380 (3%); lowest- 230 (1%) – **Dominant phyla:** Proteobacteria (Lower abundance at 2%), Firmicutes (Higher abundance at 2%), and Actinobacteria (Higher abundance at 3%)	– **Richness index (Simpson):** Lowest- 0.11 (2%); highest- 0.32 (0%)	[26]

*Procambarus clarkii*	0, 2, and 6	60	Richness index and dominant phyla	– **Richness index (Shannon):** Highest- 2.96 (6%); lowest- 2.66 (2%) – **Richness index (ACE):** Highest- 499.67 (6%); lowest- 373.22 (0%) – **Richness index (Chao1):** Highest- 489.22 (6%); lowest- 367.00 (0%) – **Richness index (Simpson):** Highest- 0.16 (2%); lowest- 0.11 (0%) – **Dominant phyla:** Firmicutes (Higher abundance at 2%), Tenericutes (Higher abundance at 2%), and Bacteroidetes (Higher abundance at 2%)	– **Dominant phyla:** Proteobacteria (Lower abundance at 0% and higher at 6%),	[44]

*Litopenaeus vannamei* ^a,b^	4	28	Richness index and dominant phyla	– **Richness index (Shannon):** Highest- 3.8 (4% KO); lowest- 2.4 (4% SL) – **Richness index (ACE):** Highest- 510 (4% KO); lowest- 350 (0%) – **Richness index (Chao1):** Highest- 510 (4% KO); lowest- 350 (0%) – **Richness index (Simpson):** Highest- 0.8 (4% KO); lowest- 0.53 (4% SL) – **Dominant phyla**: Firmicutes (Higher abundance at 4% KO) and Bacteroidetes (Higher abundance at 4% KO)	– **Dominant phyla**: Proteobacteria (Lower abundance at 4% KO),	[5]

*Note:* All diets were formulated using soy lecithin. Superscript alphabet indicates additional phospholipids sources ^a^egg yolk lecithin; ^b^krill oil lecithin. 0% refers to the control of the feeding trials.

## Data Availability

This is a review article and does not contain any primary data. All data discussed are available from the original sources cited in the manuscript.
